# Increasing climatic sensitivity of global grassland vegetation biomass and species diversity correlates with water availability

**DOI:** 10.1111/nph.17269

**Published:** 2021-03-19

**Authors:** Daijun Liu, Chao Zhang, Romà Ogaya, Marcos Fernández‐Martínez, Thomas A. M. Pugh, Josep Peñuelas

**Affiliations:** ^1^ Department of Botany and Biodiversity Research University of Vienna Rennweg 14 Vienna 1030 Austria; ^2^ School of Geography, Earth and Environmental Sciences University of Birmingham Birmingham, B15 2TT UK; ^3^ Birmingham Institute of Forest Research University of Birmingham Birmingham, B15 2TT UK; ^4^ CSIC Global Ecology Unit CREAF‐CSIC‐Universitat Autònoma de Barcelona Bellaterra (Catalonia) 08193 Spain; ^5^ Optics of Photosynthesis Laboratory Institute for Atmospheric and Earth System Research (INAR)/Forest Sciences Viikki Plant Science Centre University of Helsinki PO Box 27 Helsinki 00014 Finland; ^6^ CREAF Cerdanyola del Vallès (Catalonia) 08193 Spain; ^7^ PLECO (Plants and Ecosystems) Department of Biology University of Antwerp Wilrijk 2610 Belgium; ^8^ Department of Physical Geography and Ecosystem Science Lund University Lund 22362 Sweden

**Keywords:** carbon storage, ecological sensitivity, global warming, precipitation alteration, structural changes, temporal dynamics

## Abstract

Grasslands are key repositories of biodiversity and carbon storage and are heavily impacted by effects of global warming and changes in precipitation regimes. Patterns of grassland dynamics associated with variability in future climate conditions across spatiotemporal scales are yet to be adequately quantified. Here, we performed a global meta‐analysis of year and growing season sensitivities of vegetation aboveground biomass (AGB), aboveground net primary productivity (ANPP), and species richness (SR) and diversity (Shannon index, H) to experimental climate warming and precipitation shifts. All four variables were sensitive to climate change. Their sensitivities to shifts in precipitation were correlated with local background water availability, such as mean annual precipitation (MAP) and aridity, and AGB and ANPP sensitivities were greater in dry habitats than in nonwater‐limited habitats. There was no effect of duration of experiment (short vs long term) on sensitivities. Temporal trends in ANPP and SR sensitivity depended on local water availability; ANPP sensitivity to warming increased over time and SR sensitivity to irrigation decreased over time. Our results provide a global overview of the sensitivities of grassland function and diversity to climate change that will improve the understanding of ecological responses across spatiotemporal scales and inform policies for conservation in dry climates.

## Introduction

Ongoing global climate change, characterised by warming and spatiotemporal shifts in patterns of precipitation, is affecting species diversity and composition, and plant carbon accumulations across communities and ecosystems (Hooper *et al*., 2012; Scheffers *et al*., 2016; Thakur *et al*., 2017; Nolan *et al*., 2018). These changes in temperature and precipitation are likely to increase in both frequency and intensity in the coming decades, during which warmer and drier climates are predicted to prevail across large areas of the globe, with conditions at higher latitudes likely to become wetter (Peñuelas *et al*., 2017; Hoegh‐Guldberg *et al*., 2018; Nolan *et al*., 2018). Studies have shown that rises in carbon dioxide (CO_2_) emissions have led to increasing levels of biosphere productivity, and warmer temperatures may further increase productivity in cold regions by enhancing photosynthesis at the regional scale (Fernández‐Martínez *et al*., 2019). However, decreases in vegetation growth and reductions in terrestrial carbon uptake have also been observed and are projected to continue under future climate change scenarios, alongside increases in water limitation and nutrient mineralisation (Reich & Hobbie, 2013; Peñuelas *et al*., 2017; Fernández‐Martínez *et al*., 2019), large losses in species diversity, and disruptions to community assemblage. These impacts may be especially severe under the high‐emission RCP 8.5 (representative concentration pathway) scenario (Nolan *et al*., 2018; Berdugo *et al*., 2020; Trisos *et al*., 2020).

However, many of these predicted biodiversity and ecosystem function responses to future climate change are inconsistent with empirical evidence from natural ecosystems (Vellend *et al*., 2013; Estiarte *et al*., 2016; Messier *et al*., 2017). For example, plant growth may be regulated by plant acclimation and plasticity in physiology, phenology, and evolutionary adaptation, and may resist and recover from environmental disturbance (Jump & Peñuelas, 2005; Beier *et al*., 2012; Morecroft *et al*., 2019). Furthermore, species interactions (e.g. facilitation) may reduce the magnitude of species diversity loss and biomass declines due to positive impacts of belowground mycorrhizal and rhizobacteria associations (Wright *et al*., 2017). Climate mitigation and sustainable policies based on model predictions of biomass and species diversity declines that do not reflect real‐world responses of plant communities and ecosystems to climate change may lack effectiveness. Therefore, the development of approaches that fully detect and reflect the impacts of climate change on species diversity and function for natural ecosystems are urgently required.

Climate manipulation experiments, in which temperature and/or precipitation are controlled, are essential for the elucidation of plant adaptation and species interaction responses and mechanisms, and they reduce degrees of uncertainty of vegetation dynamics under specific climate scenarios (Jentsch *et al*., 2007; Wu *et al*., 2011; Beier *et al*., 2012; Langley *et al*., 2018). For example, empirical experimental data show contrasting spatiotemporal patterns and directions in plant community responses to climate change (Andresen *et al*., 2016; Estiarte *et al*., 2016; Harrison *et al*., 2020), such as increases in biomass accumulation at high latitudes and altitudes (Elmendorf *et al*., 2012a,b, 2015; Metcalfe *et al*., 2018) and decreases in plant growth under dry and arid conditions (Kröel‐Dulay *et al*., 2015; Ladrón de Guevara *et al*., 2018; Liu *et al*., 2020). Although changes in species diversity, richness, and composition are expected with increasing temperature and greater variability in precipitation (Knapp *et al*., 2002; Smith *et al*., 2009; Komatsu *et al*., 2019), the temporal patterns of change are not well understood. While some long‐term studies report a lack of species diversity losses under decadal climate manipulations in arctic tundra (Hudson & Henry, 2010; Elmendorf *et al*., 2012a) and Mediterranean shrublands (Tielbörger *et al*., 2014), others have demonstrated reordering shifts in species diversity and composition in grassland (Knapp *et al*., 2002; Harte *et al*., 2015; Shi *et al*., 2018), shrubland (Liu *et al*., 2017, 2018c, 2020), and forest (Liu *et al*., 2018b) ecosystems.

The integration and synthesis of empirical data from a range of study sites may allow the analysis of ecological responses over larger spatial scales (Kröel‐Dulay *et al*., 2015; Estiarte *et al*., 2016; Halbritter *et al*., 2020). However, statistical confidence in the extrapolation of pattern and trend data from a reduced number of study sites to regional and global scales is unclear. Meta‐analyses of size effects of warming and/or shifts in precipitation on ecosystem responses (e.g. biomass accumulation, species diversity, and ecosystem respiration) that have used the ln or log ratio response of treatments to that of controls (Wu *et al*., 2011; DeMalach *et al*., 2017) may hinder the comparison of response variables under contrasting magnitudes of change in simulated climate conditions, leading to uncertainty of predicted ecosystem dynamics.

Assessment of the sensitivity of vegetation responses, such as change in output per unit change in input, is a promising approach to quantify ecological impacts of climate change (Huxman *et al*., 2004; Wilcox *et al*., 2015, 2017), including across space and time (Smith *et al*., 2017). While attempts have been made to determine sensitivities of aboveground net primary productivity (ANPP) and belowground net primary productivity (BNPP) to extreme drought and increases in levels of local mean annual precipitation (MAP) at study sites (Wilcox *et al*., 2015, 2017; Smith *et al*., 2017), comparison of plant communities across the study sites may be inappropriate, due to divergence in species assemblages and successional processes driven by historical land use and other types of disturbance (Reinsch *et al*., 2017; Cramer *et al*., 2018; Langley *et al*., 2018; Liu *et al*., 2020). A novel metric of sensitivity to changes in climate, which calculates the proportional change in vegetation response per unit net change in climate and standardises response variables across study sites, has been proposed for global grasslands, desert, and forests (Song *et al*., 2019). It allows the accurate quantification and comparison of climate change studies of plant community dynamics, including biomass accumulation, and species diversity and composition, and analysis of global‐scale vegetation sensitivity across spatiotemporal gradients (Halbritter *et al*., 2020).

Grassland ecosystems are one of the largest terrestrial biomes by area (occupying *c*. 40% of the Earth’s surface) (Gibson, 2009) and are crucially important for carbon storage and biodiversity, yet are highly vulnerable to climate perturbations (Seddon *et al*., 2016; Yuan *et al*., 2016; Hungate *et al*., 2017; Wang *et al*., 2019). Application of the novel metric of Song *et al*. (2019) to data from empirical studies has indicated that sensitivities of carbon‐cycling variables in desert, wetland, grassland and forest ecosystems, such as aridity, are strongly associated with study site water availability. However, it is unclear whether similar associations of local water availability with ecosystem function and with species diversity sensitivity exist at individual grassland sites.

Temporal variation in directions of effect size (increase, decrease, no net response) have been reported (Leuzinger *et al*., 2011; Liu *et al*., 2017; Komatsu *et al*., 2019) and tend to be related to habitat factors, such water or nutrient availability (Grime *et al*., 2008; Tielbörger *et al*., 2014; Andresen *et al*., 2016). However, the ability to identify temporal trends in grassland plant growth and species diversity responses are likely to depend on the duration of an experiment (Grime *et al*., 2008; Leuzinger *et al*., 2011; Liu *et al*., 2018a), as reported for the dampening effects on growth while higher species diversity and composition shifts over time (Leuzinger *et al*., 2011; Beier *et al*., 2012; Liu *et al*., 2020). While an understanding of temporal trends in sensitivity remains lacking, possibly due to insufficient analysis of long‐term data, it is likely that site water availability may also be a key driver.

Although several global meta‐analyses have been conducted to date, how grassland sensitivity changes across spatiotemporal scales remains uncertain. Most of the previous meta‐analyses (such as Komatsu *et al*., 2019, Song *et al*., 2019 and Wu *et al*., 2011) analysed a mixture of terrestrial ecosystems (shrublands, grasslands and forests), which may have led to some uncertainties on their results. The impacts of climate change not only on the responses of ANPP and AGB, but also on species richness (SR) and community composition (Shannon index, H), were not included in most previous meta‐analyses (Wilcox *et al*., 2017; Wang *et al*., 2019). A recent study has reported the effect on SR over time, but for mixed biomes and global ecological drivers (Komatsu *et al*., 2019). To address these unknowns, we conducted a global synthesis of the effects of warming, drought, and irrigation on plant responses using a large dataset of recently published grassland experiments (138 study sites) with durations ranging from 1 to 23 yr. We tested the hypotheses that the sensitivities of grassland function and diversity to climate change are significant, the variability is affected by the local climate and magnitude of sensitivity is related to duration of experimental period. Using the dataset, the specific objectives were to assess: (1) the mean sensitivities of AGB, ANPP, SR and H to warming, drought, and irrigation; (2) the strength of associations between sensitivities and local climate conditions across spatial gradients; and (3) temporal trends in sensitivity under continued climate manipulation. We therefore aim to advance the understanding of spatiotemporal sensitivities and vulnerabilities of global grassland structure and function to climate change and to inform and improve mitigation strategies.

## Materials and Methods

### Data collection

**Fig. 1:**
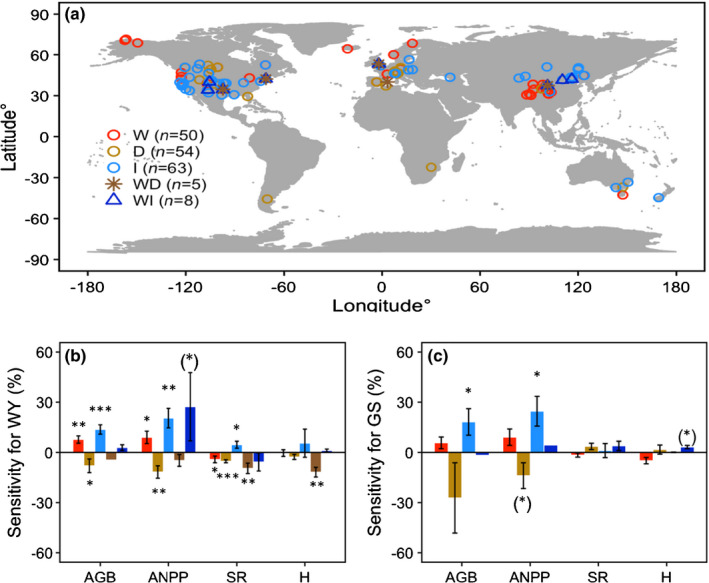
Study sites and analyses of sensitivity to climate treatments. (a) Distribution of grassland ecosystem study sites testing effects of warming (*n* = 50), drought (*n* = 54), irrigation (*n* = 63), warming and drought (*n* = 5) and warming and irrigation (*n* = 8). Colours represent the climate treatments: W, warming; D, drought; I, irrigation; WD, warming + drought; and WI, warming + irrigation. Sensitivity of aboveground biomass (AGB), aboveground net primary productivity (ANPP), species richness (SR) and species diversity (H) to climate treatments for whole years (WY) (b) and growing seasons (GS) (c). Error bars represent the standard error. The significance was tested by weighted Student's *t*‐tests ((*), *P* < 0.1; *, *P* < 0.05; **, *P* < 0.01; and ***, *P* < 0.001).

First, we searched the Web of Science database for studies published between January 1980 and April 2019 using the following single and combined keywords for manipulated climate and vegetation responses: ‘experiment*’, ‘treatment*’, ‘warm*’, ‘increase temperature’, ‘drought*’, ‘rainfall reduction’, ‘decrease precipitation’, ‘watering*’, ‘irrigation*’, ‘precipitation*’, ‘rainfall addition’, ‘biomass’, ‘growth’, ‘productivity’, ‘production’, ‘ANPP’, ‘richness’, ‘diversity’, ‘sensitivity’, ‘community’, ‘composition shifts’, ‘herb’, and ‘grass’. We also searched Google Scholar using the Advanced search function for the keywords ‘experiment*’, ‘manipulation*’, ‘biomass’, ‘grass’, ‘diversity’, ‘richness’, and ‘composition’ in the title of the article. These terms were used to identify responses of the aboveground biomass (AGB) (the standing biomass), ANPP (the net accumulation rate of aboveground biomass plus litterfall), SR and diversity (as indicated by the Shannon index H) to changes in climate (warming, and decreases and increases in precipitation). We selected climate manipulation data from recent peer‐reviewed publications (Wu *et al*., 2011; Andresen *et al*., 2016; Wilcox *et al*., 2017; Song *et al*., 2019) and datasets (https://drought‐net.colostate.edu/). We selected and used the most recently published papers on the topics and cross‐checked experimental sites, using the Advanced search function of Google Scholar, to acquire additional publications that described study site vegetation variables. We mainly focused on AGB or ANPP measured from annual harvests of global grassland plant communities and we included studies of climatic experiments conducted in ecosystems by a single factor, such as grazing or fire; multiple experiments of different vegetation communities at the same location (latitude and longitude) were recorded as a single study site. As a result of these criteria, we acquired data from 138 study sites, comprising 50 warming, 54 drought, and 63 irrigation (or water addition) single treatments, with five warming × drought and eight warming × irrigation treatment interactions. For warming experiments, we considered the impacts of elevated temperature compared with control plots, irrespective of manipulation method, such as open top chamber (OTC), cable, infrared and reflector. Data were mostly derived from experiments conducted in North America, Europe and China (Fig. 1a; Supporting Information Table S1).

When possible, we acquired vegetation dynamics and climate data from the published data sets associated with the papers; alternatively, we extracted data from figures using webplotdigitizer (v.4.2, 2019; https://automeris.io/WebPlotDigitizer) and averaged control and simulated plot data across the study period for each study site. We mainly focused on net changes in manipulated climates on species diversity and function of plant communities, so responses of variables to climate manipulations for whole years (WY) and growing seasons (GS) were treated differently. For warming, drought and irrigation treatments over WYs, we derived data for 72, 84 and 97 plant communities, respectively, while for GS, we derived data for 28, 25 and 38 communities, respectively. We used study site mean annual temperature (MAT) and ground surface temperature during the growing season from warming experiments; if air temperature was not reported, we used soil temperature (5–10 cm depth) of warming and control plots, because the temperatures of shallow soil and surface air at < 10 cm height tend to be similar (Reinsch *et al*., 2017). Local abiotic factors, comprising MAT, MAP, and growing season temperature and precipitation (Tgs and Pgs, respectively) were obtained from the Methods and Materials sections of the studies; if these data were lacking, we used control plot means. To avoid negative temperatures, we transformed MAT and Tgs (+ 15°C; MATm and Tgsm, respectively) to allow us to develop logistic regression models for the study sites. When MAT and/or MAP were not reported (e.g. Toolik lake, Finse, Haibei, Hongyuan, Beiluhe), they were derived from WorldClim (http://worldclim.org/version2, resolution *c*. 1 km^2^) that contains mean climate data for the period 1970–2000 (Fick & Hijmans, 2017). The aridity index (AI) for all the study sites was obtained from Global Aridity and PET Dataset (https://cgiarcsi.community/data/global‐aridity‐and‐pet‐database/), which provides local water availability (*c*. 1 km resolution) considering both evapotranspiration processes and rainfall deficits (Zomer *et al*., 2008).

### Sensitivity calculation and analysis

We modified the calculation of community sensitivities from previous studies (Wu *et al*., 2011; Smith *et al*., 2017; Wilcox *et al*., 2017; Song *et al*., 2019) as the plant communities across the different study sites may diverge in species assemblages and successional stages. Sensitivity was calculated as the proportion (%) of magnitude of community metric (AGB, ANPP, SR, and H) to net change in temperature [(*X*_t_ − *X*_c_)/*X*_c_)/(*T*_t_ − *T*_c_) × 100] and precipitation [(*X*_t_ − *X*_c_)/*X*_c_)/(*P*_t_ − *P*_c_) × 100], where *X*_t_ and *X*_c_ are mean AGB, ANPP, SR and H across all treatment and control years; *T*_t_ and *P*_t_ are annual mean temperature (or mean temperature of the growing season, Tgs) and annual mean precipitation (or mean precipitation of the growing season, Pgs) for treatment plots across the study period, respectively; and, *T*_c_ and *P*_c_ are annual mean temperature (or mean temperature of the growing season, Tgs) and annual mean precipitation (or precipitation of growing season, Pgs) for control plots across the study period, respectively. Therefore, the sensitivity for warming was expressed as the proportion of vegetation response per degree (%/+°C). The sensitivity for drought and irrigation treatments were expressed as the proportion of vegetation response per 100 mm change in precipitation (%/±100 mm). Sensitivities to warming × drought and warming × irrigation interactions were calculated as the proportion of vegetation response per degree per 100 mm change in precipitation or irrigation (%/+°C ± 100 mm). Positive and negative sensitivities indicated increasing and decreasing effects of climate change, respectively.

Across sites, we analysed the sensitivity of AGB, ANPP, SR and H to changes in climate, while controlling for local climate conditions using linear regressions. We did not analyse the sensitivities to warming × drought or warming × irrigation across spatial scales due to lack of available data. We tested year and growing season sensitivities of AGB and ANPP to climate change treatments separately using local MATm, MAP and AI. Correlations between treatments and SR and H were only analysed for WYs due to lack of growing season data, while those for AGB and ANPP were tested by year and growing season. Based on definitions by Knapp *et al*. (2015), we separated climate conditions into dry (MAP < 500 mm) and nonwater limited (MAP ≥ 500 mm) to test for regulation of sensitivities by site water availability.

### Definition of temporal trends

Based on duration of global climate manipulation experiments (Beier *et al*., 2012), we classified experimental temporal scales as short term (1–4 yr) or long term (≥5 yr), and selected long‐term experiments with ≥ 4 yr measurements to analyse temporal trends in ANPP and SR sensitivity; there were insufficient data for temporal analysis of H sensitivity. When climate data in control and treatments were available for study sites, we used net changes in temperature and precipitation during the study year; alternatively, we used mean magnitudes of climate change across study periods as clarified in the published literature. Temporal trends in ANPP and SR sensitivity (SEN) to climate manipulation treatments were corrected by differences between single‐year sensitivity and the mean sensitivity of each community across all years (SEN = SEN_i_ − SEN_mean_, where SEN_i_ and SEN_mean_ are sensitivity for a specific year and mean sensitivity across all years, respectively), to allow linear regression analysis of temporal sensitivity among the communities, using a reduced range of variables.

### Statistical analyses

Climate treatment effects on year and growing season AGB, ANPP, SR, and H were analysed using weighted Student's *t*‐tests in the R weights package (Pasek *et al*., 2020) to test degree of variance in average sensitivity to treatments from 0, with study year as a weighting factor to improve precision of estimated sensitivity. We then tested for associations between year and growing season sensitivities of AGB, ANPP, SR and H with habitat factors, using linear models (lm function in R), testing for treatment effects on MATm, MAP and AI for WYs and those on Tgs and Pgs for growing season; vegetation metrics were response variables and climate factors were explanatory variables. We selected the best‐fitting models based on the lowest Akaike information criterion (AIC) and considered models with an AIC within two units of the lowest value. Habitat type differences (dry vs nonwater limited) and temporal differences (short term vs long term) in sensitivities were tested using analysis of variance (ANOVA), with Tukey's honest significant difference (HSD) tests; we analysed temporal trends in ANPP and SR sensitivity across the duration of study programmes, with sensitivity as the response variable and year as the explanatory variable. We used linear mixed modelling (lme4 package in R) to test overall trends in sensitivities, with study site as a random factor. The trends for individual communities were tested using simple linear modelling. All analyses were performed in R (v.3.5.0; R Core Team, 2018).

## Results

### Sensitivity of vegetation to climate change

Vegetation function and species diversity were sensitive to climate treatments (Fig. 1b,c; Table S2), where AGB and ANPP were most sensitive. Over WYs, results showed positive response under climate warming (mean = 7.7, *P* < 0.01 and mean = 9.0, *P* < 0.05, respectively) and irrigation (mean = 13.7, *P* < 0.001 and mean = 20.5, *P* < 0.01, respectively) and negative response under drought conditions (mean = −8.0, *P* < 0.05 and mean = −11.7, *P* < 0.01, respectively) (Fig. 1b). Sensitivity of SR was negative under climate warming (mean = −4.2, *P* < 0.05), drought conditions (mean = −5.3, *P* < 0.001) and the interaction of warming × drought (mean = −9.5, *P* < 0.05), whereas it was positive under irrigation (mean = 4.6, *P* < 0.05). There were no effects of warming or drought on H sensitivity over WYs, but sensitivity was significantly negative in the interaction of warming and drought (mean = −11.8, *P* < 0.01). Growing season sensitivities of vegetation growth and community composition were affected by climate treatments (Fig. 1c), where AGB and ANPP sensitivity were positive under irrigation (mean = 18.2, *P* < 0.05 and mean = 24.6, *P* < 0.05, respectively). No influences of climate treatments on SR and H sensitivity were significant at the 5% level.

### Sensitivity of AGB and ANPP to site characteristics

**Fig. 2:**
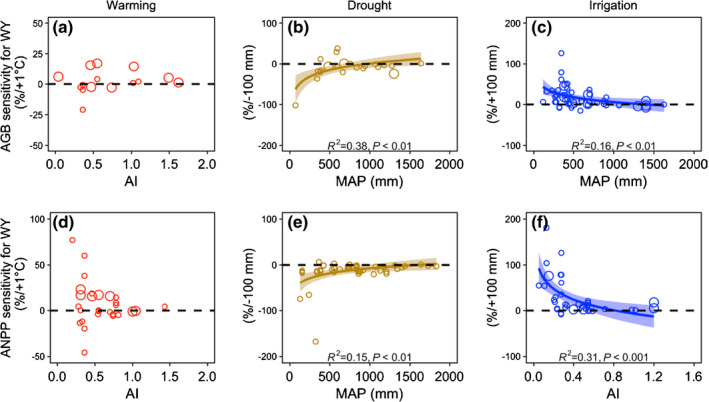
Correlations between aboveground biomass (AGB) (a–c) and aboveground net primary production (ANPP) (d–f) sensitivity to treatments over whole‐year and local climate conditions. AI and MAP represent the aridity index and mean annual precipitation of the study sites. The x‐axis shows the best‐fitted climate variables based on model selection; *R* ^2^ is the coefficient of determination for the regression. Circle sizes (small and large) represent short‐term (1–4 yr) and long‐term (≥5 yr) studies, respectively. Statistical significance across spatial scales was tested using a general linear model and only the significant relationships (*P* < 0.05) are shown (brown and blue lines). The shading with the lines indicates the standard error. The dashed line (*y* = 0) separates positive and negative values.

Over WYs, neither AGB nor ANPP sensitivity to experimental climate warming was correlated with local climate conditions (Fig. 2a,d; Table S3). WY AGB sensitivity to warming was marginally higher at dry sites than at nonwater‐limited study sites (df_HSD_ = 8.2, *P* < 0.1; Table S4) and overall temporal variation in AGB sensitivity increased with duration of experiment (df_HSD_ = −9.0, *P* < 0.05; Table S5). Growing season ANPP sensitivity to climate warming was negatively correlated with Tgsm (Fig. S1a; *R* ^2^ = 0.53, *P* < 0.05).

**Fig. 3:**
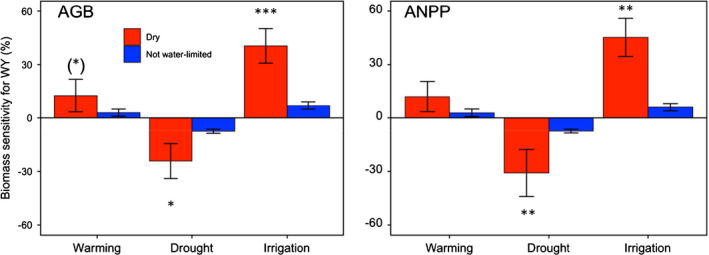
Comparison of aboveground biomass (AGB) and aboveground net primary production (ANPP) sensitivity for whole year (WY) to treatments at dry (mean annual precipitation: MAP < 500 mm) and nonwater‐limited (MAP > 500 mm) study sites, tested using analyses of variance and Tukey's honest significant difference test. Error bars represent the standard error. The asterisk indicates the different significance analysed with Tukey's honest significant difference (HSD) tests ((*), *P* < 0.1; *, *P* < 0.05; **, *P* < 0.01; and ***, *P* < 0.001).

WY AGB and ANPP sensitivity to drought was positively correlated with site MAP, respectively (*R* ^2^ = 0.38, *P* < 0.01 and *R* ^2^ = 0.15, *P* < 0.01, respectively) (Fig. 2b,e; Table S3), and was more negative in dry sites than nonwater‐limited sites (df_HSD_ = −25.8, *P* < 0.05 and df_HSD_ = −23.5, *P* < 0.01; respectively). But both sensitivities were negatively correlated with local MAP and AI in irrigated sites, respectively (*R* ^2^ = 0.16, *P* < 0.01 and *R* ^2^ = 0.31, *P* < 0.001; Fig. 2c,f). Sensitivity of plant communities (AGB and ANPP) to irrigation was positive and greater at relatively dry sites than nonwater‐limited sites (df_HSD_ = 22.7, *P* < 0.01 and df_HSD_ = 39.2, *P* < 0.01; respectively; Fig. 3; Table S4). Growing season ANPP sensitivity to drought and irrigation was correlated with local Pgs and MAP (Fig. S1b,c; *R* ^2^ = 0.40, *P* < 0.021 and *R* ^2^ = 0.27, *P* < 0.05; respectively). In other words, AGB and ANPP sensitivity to changes in precipitation was correlated with the site water availability.

### Sensitivity of species diversity to study site climate factors

**Fig. 4:**
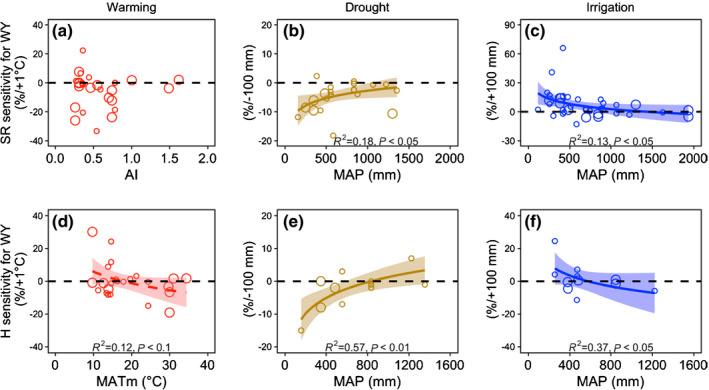
Correlations between species richness (SR) (a–c) and species diversity (H) (d–f) sensitivities for whole year (WY) under climate change treatments and local climate factors. MATm, MAP and AI represent the modified mean annual temperature (MAT + 15), mean annual precipitation and aridity index, respectively. Relationships with MATm and MAP were tested by logistic linear regression analysis; the best‐fit regressions are shown. Solid and dashed fitted regression lines indicate the significance *P* < 0.05 and *P* < 0.1, respectively, and small and large circles represent short‐term (< 5 yr) and long‐term (≥ 5 yr) experiments. The shading with the lines indicates the standard error. The dashed black line at *y* = 0 separates positive and negative values.

We did not find significant relationships between SR and H sensitivities and local climate factors under warming (Fig. 4a,d; Table S6). Under drought conditions, WY sensitivities of SR and H were positively related to local MAP (*R* ^2^ = 0.18, *P* < 0.05 and *R* ^2^ = 0.57, *P* < 0.01, respectively; Fig. 4b,e) and under irrigation, they were negatively related to local MAP (*R* ^2^ = 0.13, *P* < 0.05 and *R* ^2^ = 0.37, *P* < 0.05; respectively; Fig. 4c,f). WY sensitivity of SR under irrigation was greater in dry sites than in the nonwater‐limited sites (df_HSD_ = 12.04, *P* < 0.01) (Table S7). In other words, as for AGB and ANPP, the sensitivities of SR and H to drought and irrigation treatments were related to the site water availability.

### Temporal trends in sensitivity

**Fig. 5:**
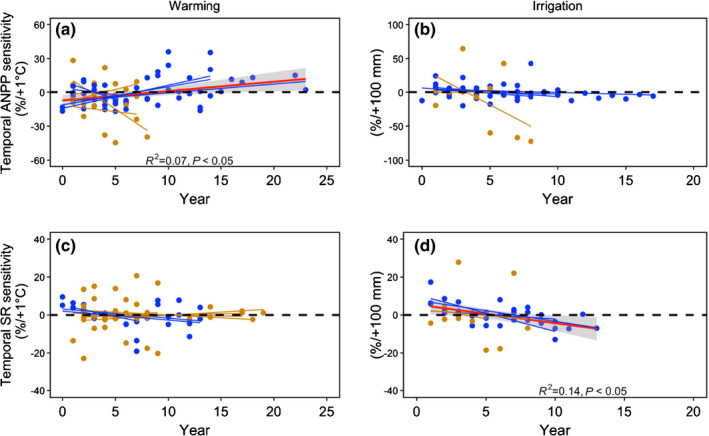
Overall temporal trends in year aboveground net primary production (ANPP) and species diversity (SR) sensitivity under experimental climate warming (a, c) and irrigation (b, d) treatment. Blue and brown dots represent nonwater‐limited (MAP > 500 mm) and dry (MAP < 500 mm) study sites, respectively. The red line (a, d) indicates the significant trend tested using a linear mixed model. The shading with the lines indicates the standard error.

We observed that the overall trend in ANPP sensitivity under climate warming increased over time (*R* ^2^ = 0.07, *P* < 0.05; Fig. 5a), but there was no such relationship for SR sensitivity (Fig. 5c). Increasing temporal ANPP sensitivity under climate warming was positively associated with study site water availability at nonwater‐limited sites (MAP = 750 mm, *R* ^2^ = 0.38, *P* < 0.01 and MAP = 914 mm, *R* ^2^ = 0.31, *P* < 0.05; Table S8), whereas SR sensitivity decreased over time at dry sites (MAP = 66.6 mm). There was no overall temporal trend in WY ANPP sensitivity under irrigated conditions (Fig. 5b), but there was a tendency for WY SR sensitivity to decrease over time (Slope = −0.9, *P* < 0.05) (Fig 5d; Table S9). There were no local climate factor effects on temporal trends in year ANPP sensitivity, however for SR sensitivity there was a temporal decrease in nonwater‐limited habitats, especially those with high levels of rainfall (Fig. 5d; MAP = 2378 mm, *R* ^2^ = 0.59, *P* < 0.01 and MAP = 700 mm, *R* ^2^ = 0.49, *P* < 0.01).

## Discussion

### Importance of quantifying vegetation sensitivity to climate change

The application of a sensitivity‐based metric is essential for the development of a global assessment of vegetation function and species diversity responses to changes in climate. To our knowledge, this study has compiled one of the largest datasets (209 plant communities across 138 study sites) for sensitivity analysis of grassland biomass and productivity (AGB and ANPP) and species diversity (SR and H) to experimental changes in temperature and precipitation. We modified the sensitivity index to calculate the vegetation response (proportion) to net climate changes, which is appropriate to compare and explore the response over spatial‐temporal scales. We found that, assessed over WYs, AGB and ANPP increased (i.e. had a positive sensitivity) under warming and increased precipitation, possibly due to enhanced biomass storage associated with elevated water and nutrient availability (Wu *et al*., 2011; DeMalach *et al*., 2017; Peñuelas *et al*., 2017; Song *et al*., 2019; Wang *et al*., 2019). However, AGB and ANPP sensitivity were negatively affected under drought conditions, probably due to constrained photosynthesis, higher levels of mortality, and reduced biomass storage (Wu *et al*., 2011; Andresen *et al*., 2016; Song *et al*., 2019; Wang *et al*., 2019). Our finding of a lack of interaction between effects of temperature and precipitation on sensitivities (Fig. 1b) was not consistent with previous research that has shown interactive effects of multiple climate factors (Komatsu *et al*., 2019). We found that SR and H were sensitive to warming and shifts in precipitation. WY SR sensitivity was negative under the single and combined treatments of warming and drought, possibly due to reduced seedling germination and establishment and ultimate local extinctions of highly sensitive species (Sullivan *et al*., 2016; LaForgia *et al*., 2018; Liu *et al*., 2018c), while WY H sensitivity was also negative under drought, indicating strong compositional shifts under harsh conditions. We observed high levels of growing season AGB and ANPP sensitivity to shifts in precipitation (Fig. 1c) that reflected the impacts of within‐season changes in precipitation on biomass accumulation and ecosystem productivity (Bai *et al*., 2004; Peñuelas *et al*., 2007, 2017; Wan *et al*., 2009). Overall, our findings, particularly those showing species diversity responses to changes in climate, have not been reported in previous studies.

### AGB and ANPP sensitivity strongly related to water availability

There were no associations between WY AGB and ANPP sensitivities under climate warming with gradients of MAT or water availability, although there was a greater number of study sites with positive sensitivities (increasing effects of warming) than negative (decreasing effects of warming) (Fig. 2a,d). Indeed, previous syntheses of large‐scale observations and climatic experiments have reported that warmer temperatures may lead to net increases in carbon assimilation and biomass storage, favouring shrub encroachment (Wu *et al*., 2011; Elmendorf *et al*., 2012a, 2015; Myers‐Smith *et al*., 2015). By contrast, WY sensitivities of AGB and ANPP to shifts in precipitation shifts were correlated with site parameters of MAP and aridity. Although these results are inconsistent with a previous study that found no relationship between grassland ANPP sensitivity and MAP under extreme drought conditions (Wilcox *et al*., 2017), our finding that AGB and ANPP sensitivities to irrigation are related to MAP and aridity is supported by previous studies (Huxman *et al*., 2004; Wilcox *et al*., 2015, 2017). Growing season ANPP sensitivities under warming were strongly correlated with study site temperature during the growing season (Tgsm) (Fig. S1). This finding has not been reported in previous studies (Wu *et al*., 2011; Song *et al*., 2019; Wang *et al*., 2019). Perhaps more importantly, we found that WY AGB and ANPP sensitivity was greater under drought and irrigated conditions at dry study sites than at nonwater‐limited sites (Fig. 3), illustrating the significant impact of increased levels of precipitation in dry habitats on biomass storage.

### SR and H sensitivity is strongly related to local climate conditions

Over WYs, sensitivities of SR and H under climate warming were not correlated with local climate conditions (Fig. 4a,d). However, under drought (Fig. 4b,e) and irrigation (Fig. 4c,d), they depended on site MAP. These results highlighted the importance of studying patterns of change in species diversity in water‐limited ecosystems (Kröel‐Dulay *et al*., 2015; Liu *et al*., 2017; Cramer *et al*., 2018). Although many studies have reported the effects of changes in climate on terrestrial carbon cycling, including ANPP and soil respiration (Jentsch *et al*., 2007; Reinsch *et al*., 2017; Song *et al*., 2019; Wang *et al*., 2019), few have quantified impacts on vegetation composition, despite overwhelming evidence for climate change‐mediated biogeographic range shifts (Lenoir *et al*., 2008; Crimmins *et al*., 2011; Rumpf *et al*., 2018), species invasion (Van Kleunen *et al*., 2015; Seebens *et al*., 2017), and shifts in dominance (Harte *et al*., 2015; Liu *et al*., 2017; Shi *et al*., 2018). High levels of global declines in SR and shifts in composition due to historical climate change have been reported, along with predictions of further large magnitudes of change in response to future climate change scenarios, especially under high levels of CO_2_ emissions (Nolan *et al*., 2018). We suggest that further research should focus on the potential impacts of climate change on species diversity and community structure, and associated impacts on higher trophic levels, species interactions and carbon cycling across different ecosystems.

### Dependence of temporal trends in AGB and SR sensitivities on local water availability

A recent meta‐analysis has reported that the effects on plant SR and community composition were greater for decadal processes (>10 yr) and when combined with drivers of global change (Komatsu *et al*., 2019). However, and with the exception of AGB sensitivity to climate warming, we did not detect differences in vegetation sensitivity to changes in climate between short‐term and long‐term studies (Table S5), possibly because the dataset comprised fewer long‐term experiments than short‐term experiments. The overall trend in ANPP sensitivity under climate warming increased over time, consistent with the expectation of an accelerated increase in storage of vegetation biomass under elevated temperatures in tundra (Elmendorf *et al*., 2012a, 2015) and alpine (Steinbauer *et al*., 2018) ecosystems. We also found that the increase in ANPP sensitivity to warming over time (Fig. 5a) was greater in high MAP sites (>700 mm), indicating water availability is a key driver of temporal patterns of biomass accumulation (Harte *et al*., 2015; Kröel‐Dulay *et al*., 2015; Estiarte *et al*., 2016; Shi *et al*., 2018). By contrast, we found that the overall trend in SR sensitivity to warming and ANPP sensitivity to irrigation did not change over time (Fig. 5c), possibly due to the plasticity of plant physiology and phenology that drive demography and species reordering (Leuzinger *et al*., 2011; Bellard *et al*., 2012; Liu *et al*., 2016; Walker *et al*., 2020) and adaptability of long‐living species as a result of functional trait compensatory responses (Smith *et al*., 2015; Sullivan *et al*., 2016). Our analysis showed an overall decreasing trend in SR sensitivity to irrigation (Fig. 5d) that was greater in study sites with high levels of water availability; these results may reflect increases in abundance and standing growth of dominant species and losses of sensitive species (Collins *et al*., 2012; Harpole *et al*., 2016; Komatsu *et al*., 2019). This study shows that temporal trends in species diversity are more sensitive at sites with high levels of water and/or nutrient availability, as supported by previous reports of lack of effects on species diversity and composition of climatic change treatments in arid (Tielbörger *et al*., 2014) and nutrient‐poor (e.g. phosphorus) (Grime *et al*., 2008) grassland ecosystems.

## Conclusion and future implications

Our global meta‐analysis quantified responses of grassland vegetation biomass and species diversity to manipulated climate conditions using the proportional change in vegetation functioning (AGB and ANPP) and species diversity (SR and H) per unit net change in manipulated temperature or precipitation. We demonstrated that this novel metric is appropriate for the quantitative assessment and comparison of ecosystem‐level responses to climate change across heterogeneous study sites and showed that vegetation sensitivity is correlated with local abiotic factors (MAP and aridity), while contrasting temporal trends in sensitivity depended on local levels of water availability. Vegetation sensitivity to climate change varied with local water availability, especially for communities distributed in dry habitats. Variability in our findings may be explained by the lack of study of species interactions, diversity and functions of soil fungi and bacteria, and nutrient cycling, and that data were derived from studies with contrasting experimental approaches (active vs passive warming), soil properties (soil types and water‐holding capacity), sample sizes and vegetation measurement protocols. Future studies should focus on the variability of sensitivity metrics to quantify impacts of climate, including patterns and timing combined with mixed drivers of climate, land use, and species invasion and the need to study the heterogeneity of fine scale abiotic and biotic habitat parameters. Therefore, broad‐scale and comprehensive evaluations of ecological sensitivities to global environmental change would further facilitate identifying generality across spatial gradients, allowing the better identification of vulnerable habitats and the design and implementation of effective protection and restoration programmes.

## Author contributions

DL and CZ developed the ideas, analysed the data and wrote the manuscript; JP and TAMP conceived the ideas and provided in‐depth suggestions for the manuscript; and, RO and MF‐M helped analyse the data and revised the manuscript.

## Supporting information


**Fig. S1**Relationships between the sensitivity of aboveground net primary production (ANPP) and habitat contexts.
**Table S1** Detailed information for the study site.
**Table S2** Results of *t*‐test for the vegetation sensitivity to experimental climate change.
**Table S3** Relationships between sensitivity of aboveground biomass (AGB) and aboveground net primary production (ANPP) in treatments for whole‐year (WY) and habitat contexts.
**Table S4** Differences between dry and nonwater‐limited sites for vegetation variables to climatic experiments.
**Table S5** Differences between short‐term and long‐term climatic experiments for the whole year.
**Table S6** Relationships between sensitivity of species richness (SR) and composition (H) to climatic change for whole‐year (WY) and habitat contexts.
**Table S7** Differences between dry and nonwater‐limited sites for vegetation variables to climatic experiments.
**Table S8** Overall and individual trend of aboveground net primary production (ANPP) and species diversity (SR) sensitivity to climate treatments over time.
**Table S9** Overall and individual trend of SR sensitivity to warming and irrigation over time.
Please note: Wiley Blackwell are not responsible for the content or functionality of any Supporting Information supplied by the authors. Any queries (other than missing material) should be directed to the *New Phytologist* Central Office.

## Data Availability

All the data and code for making graphs are available to readers upon reasonable request (d.liu@creaf.uab.es and chao.x.zhang@helsinki.fi).
