# Supplementary material for: Increasing climatic sensitivity of global grassland vegetation biomass and species diversity correlates with water availability
Source: New Phytol. 2021 Mar 19;230(5):1761–71. doi: 10.1111/nph.17269 (PMC8252445; doi:10.1111/nph.17269)
Supplement: Supplementary file 1 — Fig. S1Relationships between the sensitivity of aboveground net primary production (ANPP) and habitat contexts. Table S1 Detailed information for the study site. Table S2 Results of t‐test for the vegetation sensitivity to experimental climate change. Table S3 Relationships between sensitivity of aboveground biomass (AGB) and aboveground net primary production (ANPP) in treatments for whole‐year (WY) and habitat contexts. Table S4 Differences between dry and nonwater‐limited sites for vegetation variables to climatic experiments. Table S5 Differences between short‐term and long‐term climatic experiments for the whole year. Table S6 Relationships between sensitivity of species richness (SR) and composition (H) to climatic change for whole‐year (WY) and habitat contexts. Table S7 Differences between dry and nonwater‐limited sites for vegetation variables to climatic experiments. Table S8 Overall and individual trend of aboveground net primary production (ANPP) and species diversity (SR) sensitivity to climate treatments over time. Table S9 Overall and individual trend of SR sensitivity to warming and irrigation over time. Please note: Wiley Blackwell are not responsible for the content or functionality of any Supporting Information supplied by the authors. Any queries (other than missing material) should be directed to the New Phytologist Central Office. [file NPH-230-1761-s001.pdf]

Increasing climatic sensitivity of global grassland vegetation biomass and species diversity correlates with water availability

Daijun Liu, Chao Zhang, Romà Ogaya, Marcos Fernández-Martínez, Thomas A. M. Pugh, Josep Peñuelas

Acceptance date: 3 February 2021

Supporting Information Tables

Table S1 The detailed information for the study site.

Table S2 The result of *t* test for the vegetation sensitivity to experimental climate change.

Table S3 The relationships between sensitivity of aboveground biomass (AGB) and aboveground net primary production (ANPP) in treatments for whole year (WY) and habitat contexts.

Table S4 The differences between dry and none water-limited sites for vegetation variables to climatic experiments.

Table S5 The differences between short-term and long-term climatic experiments for whole year.

Table S6 The relationships between sensitivity of species richness (SR) and composition (H) to climatic change for whole year (WY) and habitat contexts.

Table S7 The differences between dry and none water-limited sites for vegetation variables to climatic experiments.

Table S8 The overall and individual trend of aboveground net primary production (ANPP) and species diversity (SR) sensitivity to climate treatments over time.

Table S9 The overall and individual trend of species richness (SR) sensitivity to warming and irrigation over time.

Fig. S1 The relationships between the sensitivity of aboveground net primary production (ANPP) and habitat contexts.

Table S1. The detailed information for the study site.

Country	Site	Site code	Latitude	Longitude	reference	period	treatments
Argentina	Río Mayo Experimental station	AR-PAG	-45.41	-70.16	Yahdjian & Sala (2006)	1999-2002 (3)	Drought
Australia	Tasmania	AU-TAG	-42.42	147.16	Pendall et al (2011)	2003-2007 (5)	Warming
Australia	Richmond	AU-RI	-33.36	150.44	Power et al., 2016	2013-2014 (1)	Drought, Irrigaton
Australia	Langi Ghiran State Park	AU-LGSP	-37.28	143.07	Price & morgan 2007	2001-2004 (4)	irrigation
Austrilia	Bogong	AU-BO	-36.9	147.3	Henry et al 2018	2016-2017	Drought
Canada	University of Alberta Research Ranch	CA-UARR	53.05	-111.33	Lamb et al (2007)	2004 (1)	irrigation
Canada	Great Plains	CA-GP	49.47092	-112.9403	Flanagan et al (2013)	2011 (1)	Drought, Warming, Irrigation,
Canada	Sierra Foothill Research and Extension Center	CA-SFREC	39.15	-121.17	Chou et al (2008)	2003-2006 (4)	Irrigation
Canada	Universtiy of Alberta	CA-AB	53	-111.32	White et al (2014)	2008-2009 (2)	Drought, Warming
Canada	Saskatchewan	CA-SK	49.18	-104.38	White et al (2014)	2008-2009 (2)	Drought, Warming
Canada	Manitoba	CA-MB	50.46	-100.35	White et al (2014)	2008-2009 (2)	Drought, Warming
Canada	Lac du Bois Grassland Provincial Park	CA-LBGP	50.45	-120.27	Carlyle et al (2014)	2005-2008 (4)	Drought, Irrigaton
Canada	Ontario	CA-ON	43.01	-81.12	Henry et al (2015)	2007-2013 (7)	Warming
China	Duolun	CH-DUO	42.02	116.17	Xu et al (2010;2015); Yang et al. 2018	2005-2012(8)	Irrigation
China	Duolun	CH-DUO	42.02	116.17	Yang et al (2017)	2005-2013 (9)	Warming
China	Duolun	Ch-DUO	42.02	116.17	Xiao et al (2007)	2005 (1)	irrigation
China	Duolun	Ch-DUO	42.02	116.17	Xu et al (2016)	2011-2012 (2)	warming,Irrigation
China	Haibei	CH-HAI	37.37	101.12	Klein et al (2004; 2007)	1999-2001 (3)	Warming, grazing, grazin x Warming
China	Haibei	CH-HAI	37.37	101.12	Ma et al (2017)	2011-2015 (5)	Drought, Warming, Irrigation, warming x irrigation, Warming x Drought
China	Haibei	CH-HAI	37.37	101.12	Wang et al (2012)	2006-2010 (5)	Warming
China	Damao	CH-DA	41.38	110.19	Hou et al (2013);Xu et al (2016)	2011-2012 (2)	warming,Irrigation
China	Fenghuoshan	CH-FENG	34.43	92.53	Yang et al (2015)	2006-2010 (5)	Warming
China	Songnen Grassland Ecological Reserch	CH-SGER	44.45	123.45	Zhang et al (2016)	2006-2009 (4)	Warming
China	Hongyuan	CH-HONG	31.5	101.51	Li et al (2011)	2007-2009 (3)	Warming
China	Hongyuan	CH-HONG	32.48	102.33	Zhang et al (2017)	2015-2017 (2)	Drought, Irrigaton
China	Nagqu	CH-NGQ	31.441	92.017	Ganjurjav et al (2016)	2012-2014 (3)	Warming
China	Baingoin	CH-BAIN	31.389	90.028	Ganjurjav et al (2016)	2012-2014 (3)	Warming
China	Grassland Ecological Research Station of Northeast Normal University	CH-GERS	44.45	123.45	Zhu et al (2014)	2007-2009 (3)	Drought, Irrigaton
China	Xilin river basin	CH-XRB	43.38	116.42	Gao et al (2011)	2006 (1)	Irrigation
China	Xilin river basin	CH-XRB	43.26	115.32	Brueck et al (2010)	2006 (1)	Irrigation
China	Xilin	CH-XRB	43.32	116.4	Hao et al (2017)	2012-2014(3)	Irrigation
China	Grassland Ecosystem Research Station	CH-GERS	43.38	116.42	Lü et al (2012)	2007-2008 (2)	Irrigation
China	Hulunber Grassland Ecosystem Observation	CH-HGEO	49.19	120.02	Ma et al (2012), chen et al 2018	2010-2014(5)	Irrigation
China	Changling	CH-CHANG	44.8	123.8	Henry et al 2018	2016-2017	Drought
China	Changling	CH-CHANG	44.8	123.8	Wang et al (2018)	2012-2014 (2)	irrigation
China	Beiluhe	CH-BEI	34.49	92.56	Peng et al (2014)	2012-2013(2)	Warming

China	Inner Mongolia Grassland Ecosystem Research Station	CH-ESRS	43.38	116.42	Lu et al 2018	2007-2008 (2)	irrigation
China	Bayinbuluk Grassland Ecosystem Research Station	CH-ESRS	42.88	83.7	Li et al 2015	2009-2012 (4)	irrigation
China	Gurbantunggut	CH-GUR	44.22	87.55	Zhao et al 2013	2009-2011 (3)	irrigation
china	Damxung	CH-DAM	30.25	91.05	Zong et al (2013)	2012 (1)	Warming
china	Damxung	CH-DAM	30.25	91.05	Fu et al 2013	2012 (1)	Warming
china	Songpan	CH-SONG	32.51	103.33	Shi et al (2008)	2006-2008 (2)	Warming
china	Maqu	CH-MA	33.55	101.51	Yang et al 2018	3	Warming
China	xianzha	CH-XIAN	30.57	88.42	Yang et al 2018	3	Warming
China	Guoluo	CH-GUO	34.17	100.26	Yang et al 2018	2	Warming
China	Nam Co	CH-NAM	30.43	91.03	Yang et al 2018	4	Warming
China	Qinghaihu	CH-QING	36.59	99.5	Yang et al 2018	2	Warming
China	Beiluhe	CH-BEI	34.49	92.56	Yang et al 2018	4	Warming
China	Suli	CH-SU	38.25	98.18	Yang et al 2018	2	Warming
Czech Republic	Podyji National Park	CR-PNP	48.49	16	Holub et al (2013)	2006-2007 (3)	Drought, Irrigaton
French	Massif	FR-MAG	45.47	3.05	Bloor et al (2010); Cantarel et al (2013)	2005-2009 (5)	Warming
Germany	Jena	GE-Jena	50.55	11.35			Drought
Germany	Bayreuth	GER-BA	49.9	11.6	Henry et al 2018	2016-2017	Drought
Germany	Bayreuth	GER-BA	49.55	11.34	Grant et al 2017	2008-2012 (5)	Warming
Germany	Freiburg	GER-FR	48	7.8	Henry et al 2018	2015-2016	Drought
Germany	Hiddensee	GER-HID	54.6	13.1	Henry et al 2018	2015-2016	Drought
Iceland	Thingvellir	IC-THI	64.17	21.05	Jónsdóttir et al (2005)	1995-2000 (6)	Warming
Isrral	Sde Boqer	Isral-SB	30.52	34.46	Tielbörger et al (2014)	2003-20011	Drought, Irrigaton
Isrral	Lahav	Isral-LA	31.23	34.54	Tielbörger et al (2014)	2003-20011 (9)	Drought, Irrigaton
Mogolia	Bayan Unjuul	MO-Bayan	47.02	105.57	Shinoda et al (2010)	2005 (1)	Drought
Mongolia	Dalbay Valley Lake Hovsgol	Mon-DVLH	51.01	100.45	Spence et al (2016)	2009-2012 (4)	irrigation
New Zealand	Hawea Glacial Advance	NZ-HGA	-44.68	169.18	Walker et al 2003	1988-2000 (12)	irrigation
Russia	Teberda	RU-TEB	43.27	41.41	Soudzilovskaia & Onipchenko (2005); Onipchenko et al (2012)	1998-2002 (5)	irrigation
South Africa	Kruger	SA-KR	-22.25	30.50	Koerner & Collins (2014)	2009-2011 (3)	Drought; frequent fire, frequent fire x Drought
Spain	Tabernas desert	SP-TD	37.5	2.21	Miranda et al (2011)	2009 (1)	Drought
Spain	Aranjuez Experimental Station	SP-AES	40.02	3.32	Ladrón de Guevara et al (2018)	2008-2016 (8)	Drought, Warming
Sweden	LatnjaJaure	SW-LAT	68.21	18.29	Molau (2010); Alatalo et al (2014)	1995-2006 (12)	Warming
Sweden	Great Alvar	SW-GA	56.38	16.45	Huber 1994	1985-1986 (2)	irrigation
Switzerland	Chamau	SW-CHA	47.12	8.24	Gilgen & Buchmann (2009)	2006-2007 (3)	Drought
Switzerland	Früebüel	SW-FRU	47.6	8.32	Gilgen & Buchmann (2009)	2006-2007 (3)	Drought
Switzerland	Alp Weissenstein	SW-AW	46.34	9.47	Gilgen & Buchmann (2009)	2006-2007 (2)	Drought
Switzerland	Chamau	SW-CHA	47.12	8.24	Prechsl et al (2015)	2009-2011 (3)	Drought
Switzerland	Alp Weissenstein	SW-AWS	46.34	7.47	Prechsl et al (2015)	2010-2011 (2)	Drought
Switzerland	Bister Breite	Switzerland	46.3608	8.0611	Stampfli et al (2018)	2010 (1)	Drought

Switzerland	Bister Chumme	Switzerland	46.3643	8.075	Stampfli et al (2018)	2010 (1)	Drought
Switzerland	Casserio	Switzerland	46.4415	8.9352	Stampfli et al (2018)	2010 (1)	Drought
Switzerland	Combazin	Switzerland	47.066	7.0496	Stampfli et al (2018)	2010 (1)	Drought
Switzerland	Erlenbach	Switzerland	46.6646	7.5667	Stampfli et al (2018)	2010 (1)	Drought
Switzerland	Krauchthal	Switzerland	47.0096	7.5705	Stampfli et al (2018)	2010 (1)	Drought
Switzerland	Monthey	Switzerland	47.0697	7.0627	Stampfli et al (2018)	2010 (1)	Drought
Switzerland	Negrentino	Switzerland	46.4621	8.9241	Stampfli et al (2018)	2010 (1)	Drought
Switzerland	Pree	Switzerland	45.8983	9.0094	Stampfli et al (2018)	2010 (1)	Drought
Switzerland	Somazzo	Switzerland	45.8795	8.9943	Stampfli et al (2018)	2010 (1)	Drought
Switzerland	Thun	Switzerland	46.746	7.5887	Stampfli et al (2018)	2010 (1)	Drought
Switzerland	Zollikofen	Switzerland	46.9955	7.4615	Stampfli et al (2018)	2010 (1)	Drought
Switzerland	Furka	Switzerland	46.34	8.25	De Boeck et al (2016)	2013-2015 (3)	irrigation
Switzerland	Oberwald	Switzerland	46.32	8.21	De Boeck et al (2016)	2013-2015 (3)	irrigation
Switzerland	Bister	Switzerland	46.21	8.04	De Boeck et al (2016)	2013-2015 (3)	irrigation
Switzerland	Visp	Switzerland	46.18	7.51	De Boeck et al (2016)	2013-2015 (3)	irrigation
United Kingdom	Buxton	UK-Bux	53.20	-2.00	Grime et al (2000; 2008); Morecroft et al., 2004	1994-2008 (15)	Drought, Warming, Irrigation, warming x irrigation, Warming x Drought
United Kingdom	Wytham	UK-Wy	51.46	-1.20	Grime et al (2000); Morecroft et al., 2004	1994-2001 (8)	Drought, Irrigaton
United States	Toolik Lake	USA-TL	68.38	-149.34	Hobbie & Chapin III (1998)	1991-1993 (3)	Warming
United States	Toolik Lake-inlet	USA-TLI	68.38	-149.34	Shaver et al (1998); Sistla et al (2013)	1988-2000 (14)	Warming
United States	Toolik Lake-outlet	USA-TLO	68.38	-149.34	Shaver et al (1998); Sistla et al (2013)	1988-2000 (14)	Warming
United States	Rock mountain biological laboratory (RMBL)	USA-RMBL	38.53	-107.02	Harte & Shaw (1995); Harte et al (2015)	1991-2013 (23)	Warming
United States	Oklahoma	USA-OK	34.58	-97.31	Luo et al (2009); Shi et al (2015)	2000-2014 (15)	Warming, Clipped, Clipped x Warming
United States	Atqasuk (Dry)	USA-AD	70.27	-157.24	Hollister et al (2015)	1996-2012 (17)	Warming
United States	Atqasuk (wet)	USA-AW	70.27	-157.24	Hollister et al (2015)	1996-2012 (17)	Warming
United States	Barrow (dry)	USA-BD	71.19	-156.36	Hollister et al (2015)	1994-2012 (19)	Warming
United States	Barrow (wet)	USA-BW	71.19	-156.36	Hollister et al (2015)	1995-2012 (18)	Warming
United States	Angelo Coast Range Reserve	USA-ACRR	39.44	-123.37	Suttle et al (2007); Sullivan et al (2016)	2001-2010 (10)	Irrigation
United States	Irvine Ranch Land Reserve	USA-IRLR	33.62	-117.76	Harpole et al (2007)	2004-2006 (2)	irrigation
United States	Central Plains Experimental Range (CPER)	USA-CPER	40.49	-104.46	Evans et al (2011)	1999-2009 (11)	Drought
United States	Central Plains Experimental Range (CPER)	USA-CPER	40.49	-104.46	Cherwin & Knapp (2012)	2007-2008(2)	irrigation
United States	Konza	USA-KOLG	39.05	-96.35	Collins et al (2012)	1991-2009 (19)	irrigation
United States	Konza	USA-KOUG	39.05	-96.35	Koerner & Collins (2014)	2009-2011 (3)	Drought, Clipped, Clipped x Drought
United States	Konza	USA-Konzai	39.09	-96.55	Wilcox et al (2015)	2011-2012 (2)	irrigation
United States	Konza	USA-Konza	39.05	-96.35	Fay et al (2003)	1998-2000 (3)	Drought
United States	Konza	USA-Konza	39.05	-96.35	Hoover et al (2014)	2011-2012(2)	Drought
United States	Konza	USA-Konza	39.05	-96.35	Dento et al (2017)	2013 (1)	Drought
United States	Waltham	USA-Wal	42.23	-71.12	Hoeppner & Dukes (2012)	2008-2010 (3)	Drought, Warming, Irrigation,

United States	Sevilleta National Wildlife Refuge (SNWR)	USA-SES	34.20	-106.43	Báez et al (2013)	2002-2008 (7)	Drought, Irrigaton
United States	Sevilleta National Wildlife Refuge (SNWR)	USA-SE	34.20	-106.43	Collins et al (2017)	2007-2013 (7)	Drought, Warming
United States	Sevilleta National Wildlife Refuge (SNWR)	USA-SNW	34.2	-106.43	Thomey et al (2011)	2011-2012 (2)	irrigation
United States	Boulder	USA-BOG	40.07	-105.18	Prevéy & Seastedt (2014)	2010-2013 (4)	irrigation
United States	Tenalquot	USA-TEG	46.53	-122.44	Pfeifer-Meister et al (2016)	2010-2013	Warming
United States	Willow Creek	USA-WIG	44.01	-123.10	Pfeifer-Meister et al (2016)	2010-2013	Warming
United States	Deer Creek	USA-DEG	42.16	-123.38	Pfeifer-Meister et al (2016)	2010-2013	Warming
United States	Wyoming	USA-WYG	41.11	-104.54	Morgan et al (2011), Mueller et al (2016)	2007-2013	Warming
United States	Jasper Ridge	USA-JRG	37.24	-122.14	Zhu et al (2016);Zavaleta et al. 2003	1998-2014 (17)	warming,Irrigation
United States	Irvine Ranch Conservancy	USA-IRC	33.44	-117.42	Potts et al (2012)	2006-2007 (2)	Drought, Irrigaton
United States	High Plains Grasslands Research Station	USA-HPGRS	41	-104	Chimner et al (2010)	2002-2004 (3)	Drought, Irrigaton
United States	Central Plains Experimental Range (CPER)	USA-CPER	40.49	-104.46	Byrne et al (2013)	2008-2010 (3)	Drought, Irrigaton
United States	Central Plains Experimental Range (CPER)	USA-CPER	38.52	-99.23	Byrne et al (2013)	2008-2010 (3)	Drought, Irrigaton
United States	Central Plains Experimental Range (CPER)	USA-CPER	40.84	-104.76	Wilcox et al (2015)	2011-2012 (2)	Irrigation
United States	Fort Keogh Livestock and Range Research Laboratory	USA-FKLRRL	46.31	-105.98	Wilcox et al (2015)	2011-2012 (2)	Irrigation
United States	Sand Creek Massacre	USA-SCM	38.32	-102.31	Cherwin & Knapp (2012)	2007-2008(2)	Drought
United States	Fort Union National Monument	USA-FUNM	40.49	-104.46	Cherwin & Knapp (2012)	2007-2008(2)	Drought
United States	Jasper Ridge	USA-JRG	37.40	-122.22	Henry et al (2006)	1999-2004((6)	Irrigation
United States	Chihuahuan Desert	USA-CD	32.5	-106.8	Reichmann & Sala (2014)	2007-2009 (1)	Drought, Irrigaton
United States	Kessler Farm Field Laboratory	USA-KFFL	34.58	97.31	Sherry et al (2008)	2003-2004(2)	warming,Irrigation
United States	Cheyenne	USA-CH	41.11	-104.53	Skinner et al (2002)	1997-1998(2)	Irrigation
United States	Kessler Atmospheric and Ecological Field Station	USA-KAEFS	34.59	-97.31	Xu et al (2013)	2010-2011 (2)	warming,Irrigation
United States	Kessler Atmospheric and Ecological Field Station	USA-KAEFS	34.59	-97.31	Xu et al (2013)	2010-2011 (2)	Warming, Irrigation, warming x irrigation
United States	University of California McLaughlin Reserve	USA-UCMR	38.52	-122.26	Harrision et al 2017	2015-2017 (3)	Drought, Irrigaton
United States	Hardware Ranch	USA-HR	41.6	-111.6	Henry et al 2018	2015-2016	Drought
United States	Kernen	USA-KER	52.2	-106.5	Henry et al 2018	2015-2016	Drought
United States	National Bison Range	USA-NBR	52.48	-71.36	Volence and Belovsky 2018	2014-2015 (2)	irrigation
United States	University of Florida	USA-UF	29.37	-82.21	Fahey et al 2018	2014-2018 (5)	Drought
United States	Botanical Garden of Szent István University	USA-BGSIU	40.49	-74.45	Carson & Pickett 1990	2002-2004 (3)	irrigation
United States	Miami University's Ecology Research Center	USA-MUERRC	39.5	-84.75	Stevens et al 2006	2002(1)	irrigation
United States	Sedgwick Reserve	USA-SR	34.69	-120.04	Harploe & Tilman 2007	2000-2001 (2)	irrigation
United States	Tangipahoa Parish	USA-TP	30.68	-90.48	Myers & Harms 2011	2007-2008 (2)	irrigation
United States	central Texas	USA-CT	30.51	-98.01	Wilsey et al 2014	2018-2012 (4)	irrigation
United States	Napa County	USA-NAPA	38.52	-122.26	Eskelinena & Harrison 2015	2011-2012 (2)	irrigation
United States	Niwot Ridge	USA-NR	40.05	-105.58	Winkler et al 2016	2010-2013 (4)	Warming

Publications in Table S1:

Alatalo, J. M., Jägerbrand, A. K., & Molau, U. (2014). Climate change and climatic events: community-, functional-and species-level responses of bryophytes and lichens to constant, stepwise, and pulse experimental warming in an alpine tundra. Alpine Botany, 124(2), 81-91.

Báez, S., Collins, S. L., Pockman, W. T., Johnson, J. E., & Small, E. E. (2013). Effects of experimental rainfall manipulations on Chihuahuan Desert grassland and shrubland plant communities. Oecologia, 172(4), 1117-1127.

Bloor, J. M., Pichon, P., Falcimagne, R., Leadley, P., & Soussana, J. F. (2010). Effects of warming, summer drought, and CO_2 enrichment on aboveground biomass production, flowering phenology, and community structure in an upland grassland ecosystem. Ecosystems, 13(6), 888-900.

Brueck, H., Erdle, K., Gao, Y., Giese, M., Zhao, Y., Peth, S., & Lin, S. (2010). Effects of N and water supply on water use-efficiency of a semiarid grassland in Inner Mongolia. Plant and Soil, 328(1-2), 495-505.

Byrne, K. M., Lauenroth, W. K., & Adler, P. B. (2013). Contrasting effects of precipitation manipulations on production in two sites within the central grassland region, USA. Ecosystems, 16(6), 1039-1051.

Cantarel, A. A., Bloor, J. M., & Soussana, J. F. (2013). Four years of simulated climate change reduces above-ground productivity and alters functional diversity in a grassland ecosystem. Journal of Vegetation Science, 24(1), 113-126.

Carlyle, C. N., Fraser, L. H., & Turkington, R. (2014). Response of grassland biomass production to simulated climate change and clipping along an elevation gradient. Oecologia, 174(3), 1065-1073.

Carson, W. P., & Pickett, S. T. A. (1990). Role of resources and disturbance in the organization of an old-field plant community. Ecology, 71(1), 226-238.

Chen, H., Ma, L., Xin, X., Liu, J., & Wang, R. (2018). Plant community responses to increased precipitation and belowground litter addition: Evidence from a 5-year semiarid grassland experiment. Ecology and evolution, 8(9), 4587-4597.

Cherwin, K., & Knapp, A. (2012). Unexpected patterns of sensitivity to drought in three semi-arid grasslands. Oecologia, 169(3), 845-852.

Chimner, R. A., Welker, J. M., Morgan, J., LeCain, D., & Reeder, J. (2010). Experimental manipulations of winter snow and summer rain influence ecosystem carbon cycling in a mixed-grass prairie, Wyoming, USA. Ecohydrology, 3(3), 284-293.

Chou, W. W., Silver, W. L., Jackson, R. D., Thompson, A. W., & Allen-Diaz, B. (2008). The sensitivity of annual grassland carbon cycling to the quantity and timing of rainfall. Global Change Biology, 14(6), 1382-1394.

Collins, S.L., Koerner, S.E., Plaut, J.A., Okie, J.G., Brese, D., Calabrese, L.B., Carvajal, A., Evansen, R.J. & Nonaka, E. (2012). Stability of tallgrass prairie during a 19-year increase in growing season precipitation. Functional Ecology, 26(6), 1450-1459.

Collins, S. L., Ladwig, L. M., Petrie, M. D., Jones, S. K., Mulhouse, J. M., Thibault, J. R., & Pockman, W. T. (2017). Press–pulse interactions: effects of warming, N deposition, altered winter precipitation, and fire on desert grassland community structure and dynamics. Global change biology, 23(3), 1095-1108.

De Boeck, H. J., Bassin, S., Verlinden, M., Zeiter, M., & Hiltbrunner, E. (2016). Simulated heat waves affected alpine grassland only in combination with drought. New Phytologist, 209(2), 531-541.

Denton, E. M., Dietrich, J. D., Smith, M. D., & Knapp, A. K. (2017). Drought timing differentially affects above-and belowground productivity in a mesic grassland. Plant Ecology, 218(3), 317-328.

Eskelinen, A., & Harrison, S. (2015). Biotic context and soil properties modulate native plant responses to enhanced rainfall. Annals of botany, 116(6), 963-973.

Evans, S. E., Byrne, K. M., Lauenroth, W. K., & Burke, I. C. (2011). Defining the limit to resistance in a drought-tolerant grassland: long-term severe drought significantly reduces the dominant species and increases ruderals. Journal of Ecology, 99(6), 1500-1507.

Fahey, C., Angelini, C., & Flory, S. L. (2018). Grass invasion and drought interact to alter the diversity and structure of native plant communities. Ecology, 99(12), 2692-2702.

Fay, P. A., Carlisle, J. D., Knapp, A. K., Blair, J. M., & Collins, S. L. (2003). Productivity responses to altered rainfall patterns in a C_4-dominated grassland. Oecologia, 137(2), 245-251.

Flanagan, L. B., Sharp, E. J., & Letts, M. G. (2013). Response of plant biomass and soil respiration to experimental warming and precipitation manipulation in a Northern Great Plains grassland. Agricultural and Forest Meteorology, 173, 40-52.

Fu, G., Zhang, X., Zhang, Y., Shi, P., Li, Y., Zhou, Y., Yang, P. & Shen, Z. (2013). Experimental warming does not enhance gross primary production and above-ground biomass in the alpine meadow of Tibet. Journal of Applied Remote Sensing, 7(1), 073505.

Ganjurjav, H., Gao, Q., Gornish, E.S., Schwartz, M.W., Liang, Y., Cao, X., Zhang, W., Zhang, Y., Li, W., Wan, Y. and Li, Y. (2016). Differential response of alpine steppe and alpine meadow to climate warming in the central Qinghai–Tibetan Plateau. Agricultural and Forest Meteorology, 223, 233-240.

Gao, Y. Z., Chen, Q., Lin, S., Giese, M., & Brueck, H. (2011). Resource manipulation effects on net primary production, biomass allocation and rain-use efficiency of two semiarid grassland sites in Inner Mongolia, China. Oecologia, 165(4), 855-864.

Gilgen, A. K., & Buchmann, N. (2009). Response of temperate grasslands at different altitudes to simulated summer drought differed but scaled with annual precipitation. Biogeosciences Discussions, 6(3), 5217-5250.

Grant, K., Kreyling, J., Beierkuhnlein, C., & Jentsch, A. (2017). Importance of seasonality for the response of a mesic temperate grassland to increased precipitation variability and warming. Ecosystems, 20(8), 1454-1467.

Grime, J.P., Brown, V.K., Thompson, K., Masters, G.J., Hillier, S.H., Clarke, I.P., Askew, A.P., Corker, D. & Kielty, J.P. 2000. The response of two contrasting limestone grasslands to simulated climate change. Science, 289(5480), 762-765.

Grime, J. P., Fridley, J. D., Askew, A. P., Thompson, K., Hodgson, J. G., & Bennett, C. R. (2008). Long-term resistance to simulated climate change in an infertile grassland. Proceedings of the National Academy of Sciences, 105(29), 10028-10032.

Hao, Y.B., Zhou, C.T., Liu, W.J., Li, L.F., Kang, X.M., Jiang, L.L., Cui, X.Y., Wang, Y.F., Zhou, X.Q. & Xu, C.Y. (2017). Aboveground net primary productivity and carbon balance remain stable under extreme precipitation events in a semiarid steppe ecosystem. Agricultural and Forest Meteorology, 240, 1-9.

Harpole, W. S., & Tilman, D. (2007). Grassland species loss resulting from reduced niche dimension. Nature, 446(7137), 791-793.

Harpole, W. S., Potts, D. L., & Suding, K. N. (2007). Ecosystem responses to water and nitrogen amendment in a California grassland. Global Change Biology, 13(11), 2341-2348.

Harrison, S. P., LaForgia, M. L., & Latimer, A. M. (2018). Climate-driven diversity change in annual grasslands: Drought plus deluge does not equal normal. Global change biology, 24(4), 1782-1792.

Harte, J., & Shaw, R. (1995). Shifting dominance within a montane vegetation community: results of a climate-warming experiment. Science, 267(5199), 876-880.

Harte, J., Saleska, S. R., & Levy, C. (2015). Convergent ecosystem responses to 23-year ambient and manipulated warming link advancing snowmelt and shrub encroachment to transient and long-term climate–soil carbon feedback. Global Change Biology, 21(6), 2349-2356.

Henry, H.A., Abedi, M., Alados, C.L., Beard, K.H., Fraser, L.H., Jentsch, A., Kreyling, J., Kulmatiski, A., Lamb, E.G., Sun, W. & Vankoughnett, M.R. (2018). Increased soil frost versus summer drought as drivers of plant biomass responses to reduced precipitation: results from a globally coordinated field experiment. Ecosystems, 21(7), 1432-1444.

Henry, H. A., Chiariello, N. R., Vitousek, P. M., Mooney, H. A., & Field, C. B. (2006). Interactive effects of fire, elevated carbon dioxide, nitrogen deposition, and precipitation on a California annual grassland. Ecosystems, 9(7), 1066-1075.

Henry, H. A., Hutchison, J. S., Kim, M. K., & McWhirter, B. D. (2015). Context matters for warming: interannual variation in grass biomass responses to 7 years of warming and N addition. Ecosystems, 18(1), 103-114.

Hobbie, S. E., & Chapin III, F. S. (1998). The response of tundra plant biomass, aboveground production, nitrogen, and CO_2 flux to experimental warming. Ecology, 79(5), 1526-1544.

Hoeppner, S. S., & Dukes, J. S. (2012). Interactive responses of old-field plant growth and composition to warming and precipitation. Global Change Biology, 18(5), 1754-1768.

Hollister, R.D., May, J.L., Kremers, K.S., Tweedie, C.E., Oberbauer, S.F., Liebig, J.A., Botting, T.F., Barrett, R.T. & Gregory, J.L. (2015). Warming experiments elucidate the drivers of observed directional changes in tundra vegetation. Ecology and evolution, 5(9), 1881-1895.

Holub, P., Fabšičová, M., Tůma, I., Záhora, J., & Fiala, K. (2013). Effects of artificially varying amounts of rainfall on two semi-natural grassland types. Journal of vegetation science, 24(3), 518-529.

Hoover, D. L., Knapp, A. K., & Smith, M. D. (2014). Resistance and resilience of a grassland ecosystem to climate extremes. Ecology, 95(9), 2646-2656.

Hou, Y., Zhou, G., Xu, Z., Liu, T., & Zhang, X. (2013). Interactive effects of warming and increased precipitation on community structure and composition in an annual forb dominated desert steppe. PLoS One, 8(7), e70114.

Huber, R. (1994). Changes in plant species richness in a calcareous grassland following changes in environmental conditions. Folia Geobotanica et Phytotaxonomica, 29(4), 469-482.

Jónsdóttir, I. S., Magnússon, B., Gudmundsson, J., Elmarsdóttir, Á., & Hjartarson, H. (2005). Variable sensitivity of plant communities in Iceland to experimental warming. Global Change Biology, 11(4), 553-563.

Klein, J. A., Harte, J., & Zhao, X. Q. (2004). Experimental warming causes large and rapid species loss, dampened by simulated grazing, on the Tibetan Plateau. Ecology Letters, 7(12), 1170-1179.

Klein, J. A., Harte, J., & Zhao, X. Q. (2007). Experimental warming, not grazing, decreases rangeland quality on the Tibetan Plateau. Ecological Applications, 17(2), 541-557.

Koerner, S. E., & Collins, S. L. (2014). Interactive effects of grazing, drought, and fire on grassland plant communities in North America and South Africa. Ecology, 95(1), 98-109.

Lamb, E. G., Shore, B. H., & Cahill, J. F. (2007). Water and nitrogen addition differentially impact plant competition in a native rough fescue grassland. Plant Ecology, 192(1), 21-33.

Li, G., Liu, Y., Frelich, L. E., & Sun, S. (2011). Experimental warming induces degradation of a Tibetan alpine meadow through trophic interactions. Journal of Applied Ecology, 48(3), 659-667.

Li, K., Liu, X., Song, L., Gong, Y., Lu, C., Yue, P., Tian, C. & Zhang, F. (2015). Response of alpine grassland to elevated nitrogen deposition and water supply in China. Oecologia, 177(1), 65-72.

Lü, X. T., Kong, D. L., Pan, Q. M., Simmons, M. E., & Han, X. G. (2012). Nitrogen and water availability interact to affect leaf stoichiometry in a semi-arid grassland. Oecologia, 168(2), 301-310.

Lü, X. T., Liu, Z. Y., Hu, Y. Y., & Zhang, H. Y. (2018). Testing nitrogen and water co-limitation of primary productivity in a temperate steppe. Plant and soil, 432(1-2), 119-127.

Luo, Y., Sherry, R., Zhou, X., & Wan, S. (2009). Terrestrial carbon-cycle feedback to climate warming: experimental evidence on plant regulation and impacts of biofuel feedstock harvest. GCB Bioenergy, 1(1), 62-74.

Ma, L., Huang, W., Guo, C., Wang, R., & Xiao, C. (2012). Soil microbial properties and plant growth responses to carbon and water addition in a temperate steppe: the importance of nutrient availability. PloS one, 7(4), e35165.

Ma, Z., Liu, H., Mi, Z., Zhang, Z., Wang, Y., Xu, W., Jiang, L. & He, J.S. (2017). Climate warming reduces the temporal stability of plant community biomass production. Nature Communications, 8(1), 1-7.

Miranda, J. D. D., Armas, C., Padilla, F. M., & Pugnaire, F. I. (2011). Climatic change and rainfall patterns: effects on semi-arid plant communities of the Iberian Southeast. Journal of Arid Environments, 75(12), 1302-1309.

Molau, U. (2010). Long-term impacts of observed and induced climate change on tussock tundra near its southern limit in northern Sweden. Plant Ecology & Diversity, 3(1), 29-34.

Morecroft, M. D., Masters, G. J., Brown, V. K., Clarke, I. P., Taylor, M. E., & Whitehouse, A. T. (2004). Changing precipitation patterns alter plant community dynamics and succession in an ex-arable grassland. Functional ecology, 18(5), 648-655.

Morgan, J.A., LeCain, D.R., Pendall, E., Blumenthal, D.M., Kimball, B.A., Carrillo, Y., Williams, D.G., Heisler-White, J., Dijkstra, F.A. & West, M. (2011). C_4 grasses prosper as carbon dioxide eliminates desiccation in warmed semi-arid grassland. Nature, 476(7359), 202-205.

Mueller, K. E., Blumenthal, D. M., Pendall, E., Carrillo, Y., Dijkstra, F. A., Williams, D. G., Follett, R. F., & Morgan, J. A. (2016). Impacts of warming and elevated CO_2 on a semi-arid grassland are non-additive, shift with precipitation, and reverse over time. Ecology Letters, 19(8), 956-966.

Myers, J. A., & Harms, K. E. (2011). Seed arrival and ecological filters interact to assemble high-diversity plant communities. Ecology, 92(3), 676-686.

Onipchenko, V.G., Makarov, M.I., Akhmetzhanova, A.A., Soudzilovskaia, N.A., Aibazova, F.U., Elkanova, M.K., Stogova, A.V. & Cornelissen, J.H.C. (2012). Alpine plant functional group responses to fertiliser addition depend on abiotic regime and community composition. Plant and Soil, 357(1-2), 103-115.

Pendall, E., Osanai, Y. U. I., Williams, A. L., & Hovenden, M. J. (2011). Soil carbon storage under simulated climate change is mediated by plant functional type. Global Change Biology, 17(1), 505-514.

Peng, F., You, Q., Xu, M., Guo, J., Wang, T., & Xue, X. (2014). Effects of warming and clipping on ecosystem carbon fluxes across two hydrologically contrasting years in an alpine meadow of the Qinghai-Tibet Plateau. PLoS One, 9(10), e109319.

Pfeifer-Meister, L., Bridgham, S.D., Reynolds, L.L., Goklany, M.E., Wilson, H.E., Little, C.J., Ferguson, A. & Johnson, B.R. (2016). Climate change alters plant biogeography in Mediterranean prairies along the West Coast, USA. Global change biology, 22(2), 845-855.

Potts, D. L., Suding, K. N., Winston, G. C., Rocha, A. V., & Goulden, M. L. (2012). Ecological effects of experimental drought and prescribed fire in a southern California coastal grassland. Journal of Arid Environments, 81, 59-66.

Power, S.A., Barnett, K.L., Ochoa-Hueso, R., Facey, S.L., Gibson-Forty, E.V., Hartley, S.E., Nielsen, U.N., Tissue, D.T. & Johnson, S.N. (2016). DRI-Grass: a new experimental platform for addressing grassland ecosystem responses to future precipitation scenarios in south-east Australia. Frontiers in plant science, 7, 1373.

Prechsl, U. E., Burri, S., Gilgen, A. K., Kahmen, A., & Buchmann, N. (2015). No shift to a deeper water uptake depth in response to summer drought of two lowland and sub-alpine C_3-grasslands in Switzerland. Oecologia, 177(1), 97-111.

Prevéy, J. S., & Seastedt, T. R. (2014). Seasonality of precipitation interacts with exotic species to alter composition and phenology of a semi-arid grassland. Journal of Ecology, 102(6), 1549-1561.

Price, J. N., & Morgan, J. W. (2007). Vegetation dynamics following resource manipulations in herb-rich woodland. Plant Ecology, 188(1), 29-37.

Reichmann, L. G., & Sala, O. E. (2014). Differential sensitivities of grassland structural components to changes in precipitation mediate productivity response in a desert ecosystem. Functional Ecology, 28(5), 1292-1298.

Shaver, G. R., Johnson, L. C., Cades, D. H., Murray, G., Laundre, J. A., Rastetter, E. B., Nadelhoffer, K.J. & Giblin, A. E. (1998). Biomass and CO_2 flux in wet sedge tundras: responses to nutrients, temperature, and light. Ecological Monographs, 68(1), 75-97.

Sherry, R.A., Weng, E., ARNONE III, J.A., Johnson, D.W., Schimel, D.S., Verburg, P.S., Wallace, L.L. & Luo, Y. (2008). Lagged effects of experimental warming and doubled precipitation on annual and seasonal aboveground biomass production in a tallgrass prairie. Global Change Biology, 14(12), 2923-2936.

Shi, Z., Sherry, R., Xu, X., Hararuk, O., Souza, L., Jiang, L., Xia, J., Liang, J. & Luo, Y. (2015). Evidence for long-term shift in plant community composition under decadal experimental warming. Journal of Ecology, 103(5), 1131-1140.

Shinoda, M., Nachinshonhor, G. U., & Nemoto, M. (2010). Impact of drought on vegetation dynamics of the Mongolian steppe: a field experiment. Journal of Arid Environments, 74(1), 63-69.

Sistla, S. A., Moore, J. C., Simpson, R. T., Gough, L., Shaver, G. R., & Schimel, J. P. (2013). Long-term warming restructures Arctic tundra without changing net soil carbon storage. Nature, 497(7451), 615-618.

Skinner, R. H., Hanson, J. D., Hutchinson, G. L., & Schuman, G. E. (2002). Response of C_3 and C_4 grasses to supplemental summer precipitation. Journal of Range Management, 517-522.

Soudzilovskaia, N. A., & Onipchenko, V. G. (2005). Experimental investigation of fertilization and irrigation effects on an alpine heath, northwestern Caucasus, Russia. Arctic, Antarctic, and Alpine Research, 37(4), 602-610.

Spence, L. A., Liancourt, P., Boldgiv, B., Petraitis, P. S., & Casper, B. B. (2016). Short-term manipulation of precipitation in Mongolian steppe shows vegetation influenced more by timing than amount of rainfall. Journal of Vegetation Science, 27(2), 249-258.

Stampfli, A., Bloor, J. M., Fischer, M., & Zeiter, M. (2018). High land-use intensity exacerbates shifts in grassland vegetation composition after severe experimental drought. Global change biology, 24(5), 2021-2034.

Stevens, M. H. H., Shirk, R., & Steiner, C. E. (2006). Water and fertilizer have opposite effects on plant species richness in a mesic early successional habitat. Plant Ecology, 183(1), 27-34.

Sullivan, M. J., A. Thomsen, M., & Suttle, K. B. (2016). Grassland responses to increased rainfall depend on the timescale of forcing. Global change biology, 22(4), 1655-1665.

Suttle, K. B., Thomsen, M. A., & Power, M. E. (2007). Species interactions reverse grassland responses to changing climate. Science, 315(5812), 640-642.

Thomey, M. L., Collins, S. L., Vargas, R., Johnson, J. E., Brown, R. F., Natvig, D. O., & Friggens, M. T. (2011). Effect of precipitation variability on net primary production and soil respiration in a Chihuahuan Desert grassland. Global Change Biology, 17(4), 1505-1515.

Volenec, Z. M., & Belovsky, G. E. (2018). The interaction of temperature and precipitation determines productivity and diversity in a bunchgrass prairie ecosystem. Oecologia, 188(3), 913-920.

Walker, S., Wilson, J.B. & Lee, W.G. (2003). Recovery of short tussock and woody species guilds in ungrazed Festuca novae-zelandiae short tussock grassland with fertiliser or irrigation. New Zealand Journal of Ecology, 27(2): 179-189.

Wang, S., Duan, J., Xu, G., Wang, Y., Zhang, Z., Rui, Y., Luo, C., Xu, B., Zhu, X., Chang, X. & Cui, X. (2012). Effects of warming and grazing on soil N availability, species composition, and ANPP in an alpine meadow. Ecology, 93(11), 2365-2376.

Wang, Y., Meng, B., Zhong, S., Wang, D., Ma, J., & Sun, W. (2018). Aboveground biomass and root/shoot ratio regulated drought susceptibility of ecosystem carbon exchange in a meadow steppe. Plant and Soil, 432(1-2), 259-272

White, S. R., Bork, E. W., & Cahill Jr, J. F. (2014). Direct and indirect drivers of plant diversity responses to climate and clipping across northern temperate grassland. Ecology, 95(11), 3093-3103.

Wilcox, K. R., von Fischer, J. C., Muscha, J. M., Petersen, M. K., & Knapp, A. K. (2015). Contrasting above-and belowground sensitivity of three Great Plains grasslands to altered rainfall regimes. Global Change Biology, 21(1), 335-344.

Wilsey, B. J., Daneshgar, P. P., Hofmockel, K., & Polley, H. W. (2014). Invaded grassland communities have altered stability-maintenance mechanisms but equal stability compared to native communities. Ecology Letters, 17(1), 92-100.

Winkler, D. E., Chapin, K. J., & Kueppers, L. M. (2016). Soil moisture mediates alpine life form and community productivity responses to warming. Ecology, 97(6), 1553-1563.

Xiao, C., Janssens, I. A., Liu, P., Zhou, Z., & Sun, O. J. (2007). Irrigation and enhanced soil carbon input effects on below-ground carbon cycling in semiarid temperate grasslands. New Phytologist, 174(4), 835-846.

Xu, M., Peng, F., You, Q., Guo, J., Tian, X., Xue, X., & Liu, M. (2015). Year-round warming and autumnal clipping lead to downward transport of root biomass, carbon and total nitrogen in soil of an alpine meadow. Environmental and Experimental Botany, 109, 54-62.

Xu, X., Sherry, R. A., Niu, S., Li, D., & Luo, Y. (2013). Net primary productivity and rain-use efficiency as affected by warming, altered precipitation, and clipping in a mixed-grass prairie. Global change biology, 19(9), 2753-2764.

Xu, Z., Hou, Y., Zhang, L., Liu, T., & Zhou, G. (2016). Ecosystem responses to warming and watering in typical and desert steppes. Scientific reports, 6, 34801.

Xu, Z., Wan, S., Zhu, G., Ren, H., & Han, X. (2010). The influence of historical land use and water availability on grassland restoration. Restoration Ecology, 18, 217-225.

Yahdjian, L., & Sala, O. E. (2006). Vegetation structure constrains primary production response to water availability in the Patagonian steppe. Ecology, 87(4), 952-962.

Yang, Y., Hopping, K. A., Wang, G., Chen, J., Peng, A., & Klein, J. A. (2018). Permafrost and drought regulate vulnerability of Tibetan Plateau grasslands to warming. Ecosphere, 9(5), e02233.

Yang, Y., Wang, G., Klanderud, K., Wang, J., & Liu, G. (2015). Plant community responses to five years of simulated climate warming

Yang, Z., Zhang, Q., Su, F., Zhang, C., Pu, Z., Xia, J., Wan, S. & Jiang, L. (2017). Daytime warming lowers community temporal stability by reducing the abundance of dominant, stable species. Global change biology, 23(1), 154-163.

Zavaleta, E. S., Shaw, M. R., Chiariello, N. R., Thomas, B. D., Cleland, E. E., Field, C. B., & Mooney, H. A. (2003). Grassland responses to three years of elevated temperature, CO_2, precipitation, and N deposition. Ecological monographs, 73(4), 585-604.

Zhang, F., Quan, Q., Song, B., Sun, J., Chen, Y., Zhou, Q., & Niu, S. (2017). Net primary productivity and its partitioning in response to precipitation gradient in an alpine meadow. Scientific reports, 7(1), 1-10.

Zhang, Y., Loreau, M., Lü, X., He, N., Zhang, G., & Han, X. (2016). Nitrogen enrichment weakens ecosystem stability through decreased species asynchrony and population stability in a temperate grassland. Global change biology, 22(4), 1445-1455.

Zhao, X., Xu, H., Zhang, P., Fu, J., Tu, W., & Zhang, Q. (2013). The effects of nutrient addition on plant species diversity in desert grassland, Xinjiang, northwest China. Quaternary international, 298, 152-160.

Zhu, H., Wang, D., Wang, L., Fang, J., Sun, W., & Ren, B. (2014). Effects of altered precipitation on insect community composition and structure in a meadow steppe. Ecological entomology, 39(4), 453-461.

Zhu, K., Chiariello, N. R., Tobeck, T., Fukami, T., & Field, C. B. (2016). Nonlinear, interacting responses to climate limit grassland production under global change. Proceedings of the National Academy of Sciences, 113(38), 10589-10594.

Zong, N., Shi, P., Jiang, J., Song, M., Xiong, D., Ma, W., Fu, G., Zhang, X. & Shen, Z. (2013). Responses of ecosystem CO_2 fluxes to short-term experimental warming and nitrogen enrichment in an alpine meadow, Northern Tibet Plateau. The Scientific World, 1-11, http://dx.doi.org/10.1155/2013/415318.

Shi, F., Wu, N., & Luo, P. (2008) Effect of temperature enhancement on community structure and biomass of subalpine meadow in Northwestern Sichuan. Acta Ecologica Sinica, 28(11): 5286-5293.

Table S2. The result of *t*-test for the vegetation sensitivity to experimental climate change. The treatments were separated by two types for whole year and growing season. The significance of vegetation variables of aboveground biomass (AGB), aboveground net primary production (ANPP), species richness (SR) and composition (H) in response to each treatment was separately tested from zero. The variable with significance (p<0.05) was highlighted with bold (*, p<0.05; **, p<0.01; ***, p<0.001), and (*) and ns represent p<0.1 and no significance respectively. SEN_mean, SE and N are the mean sensitivity, standard error and number of study communities.

Climate types	Treatments	Variables	SEN_mean	SE	N	*p* value
Treatment for whole year	Warming	**AGB**	**7.7**	**2.2**	**15**	**0.004****
		**ANPP**	**9**	**3.7**	**28**	**0.02***
		**SR**	**-4.2**	**2**	**27**	**0.04***
		H	-0.4	2	24	ns
	Drought	**AGB**	**-8**	**4.1**	**21**	**0.04***
		**ANPP**	**-11.7**	**3.7**	**48**	**0.003****
		**SR**	**-5.3**	**0.9**	**24**	**<0.001*****
		H	-2.7	1.5	12	ns
	Irrigation	**AGB**	**13.7**	**2.8**	**53**	**<0.001*****
		**ANPP**	**20.5**	**5.8**	**37**	**0.001****
		**SR**	**4.6**	**2.1**	**38**	**0.04***
		H	5.5	8.4	12	ns
	Warming x Drought	AGB	-4.5	-	1	-
		ANPP	-4.8	3.5	5	ns
		**SR**	**-9.5**	**3.1**	**6**	**0.03***
		**H**	**-11.8**	**2.9**	**7**	**0.006****
	Warming x Irrigation	AGB	2.9	1.7	2	ns
		ANPP	27.3	20.4	8	ns
		SR	-5.7	5.4	10	ns
		H	1.1	0.9	7	ns
Treatment for growing season	Warming	AGB	5.7	3.5	13	ns
		ANPP	9.1	4.9	9	ns
		SR	-1.6	1.2	25	ns
		**H**	**-4.9**	**1.9**	**26**	**0.02***
	Drought	AGB	-27.2	21	3	ns
		ANPP	-13.9	7.7	20	0.09(*)
		SR	3.6	1.9	11	0.09(*)
		H	1.7	2.7	3	ns
	Irrigation	**AGB**	**18.2**	**7.9**	**18**	**0.03***
		**ANPP**	**24.6**	**8.9**	**22**	**0.01***
		SR	1.1	4.2	9	ns
		H	0.09	0.12	4	ns
	Warming x Drought	AGB	-	-	-	-
		ANPP	-	-	-	-
		SR	-	-	-	-
		H	-	-	-	-
	Warming x Irrigation	AGB	-1.7	-	1	-
		ANPP	4.4	-	1	-
		SR	3.9	2.8	4	ns
		H	3.2	1	3	0.08 (*)

Table S3. The relationships between sensitivity of aboveground biomass (AGB) and aboveground net primary production (ANPP) in treatments for whole year (WY) and habitat contexts. There were three climate parameters of modified mean annual temperature (MATm), mean annual precipitation (MAP) and aridity index (AI). The statistical analysis was tested by linear regression model. The significances were highlighted and marked with asterisks (*, p<0.05 and **, p<0.01), and (*) and ns represent p<0.1 and no significance respectively.

Treatments	Variables	Habitat contexts	AIC	slope	t value	R^2 value	p value
Warming for WY	AGB	MATm	107.9	-5.9	-0.5	0.01	ns
		MAP	113.9	-2.8	-0.3	0.001	ns
		AI	114.2	0.9	0.08	0.01	ns
	ANPP	MATm	260.5	3.5	1.1	0.08	ns
		MAP	260.6	5	0.85	0.5	ns
		AI	260.8	-3.14	-0.68	0.03	ns
Drought for WY	AGB	**MATm**	**196**	**3.2**	**2.6**	**0.26**	**0.02***
		**MAP**	**193**	**24**	**3.4**	**0.38**	**0.002****
		**AI**	**195.6**	**25**	**2.8**	**0.3**	**0.02***
	ANPP	MATm	453.5	-24.9	-1.8	0.06	0.09 (*)
		**MAP**	**448.8**	**15.7**	**2.9**	**0.15**	**0.006****
		**AI**	**446.9**	**15.1**	**3.2**	**0.19**	**0.002****
Irrigation for WY	AGB	**MATm**	**486.3**	**-26.2**	**-2.5**	**0.11**	**0.02***
		**MAP**	**481.6**	**-21.1**	**-3.3**	**0.18**	**0.002****
		AI	488.6	-14.1	-1.9	0.07	0.07 (*)
	ANPP	MATm	375.8	-9	-0.4	0.001	ns
		**MAP**	**371.4**	**-0.04**	**-2.2**	**0.13**	**0.03***
		**AI**	**367.4**	**-33.9**	**-3**	**0.21**	**0.004****

Table S4. The differences between dry and none water-limited sites for vegetation variables to climatic experiments. The dry sites stand for the mean annual precipitation (MAP) less than 500 mm while none water-limited ones of MAP larger than 500 mm. The vegetation variables of aboveground biomass (AGB), Species richness (SR) and community composition (H) between the two climatic types were separately analysed by ANOVA with Turkey HSD. The significances were highlighted and marked with asterisks (*, p<0.05 and **, p<0.01), and (*) and ns represent p<0.1 and no significance respectively.

Treatments	Variables	dfHSD	*p* value
Warming for WY	AGB	8.2	0.09 (*)
	ANPP	-9.2	ns
Drought for WY	**AGB**	**-25.8**	**0.04***
	**ANPP**	**-23.5**	**0.006****
Irrigation for WY	**AGB**	**22.7**	**0.001****
	**ANPP**	**39.2**	**0.003****

Table S5. The differences between short-term and long-term climatic experiments for whole year. The short-term is less than 5 years and long-term is more than 5 years. The vegetation variables of aboveground biomass (AGB), aboveground net primary production (ANPP), species richness (SR) and community composition (H) were separately analysed by ANOVA with Turkey HSD. The significances were highlighted and marked with asterisks (*, $p<0.05$), and ns represents no significance.

Treatments	Variables	dfHSD	p value
**Warming for WY**	**AGB**	**-9**	**0.04***
	ANPP	-6.7	ns
	SR	-4.4	ns
	H	-0.5	ns
**Drought for WY**	AGB	0.07	ns
	ANPP	-	-
	SR	-4	ns
	H	-1.4	ns
**Irrigation for WY**	AGB	12.2	ns
	ANPP	12.5	ns
	SR	-4.5	ns
	H	-24.7	ns

Table S6. The relationships between sensitivity of species richness (SR) and composition (H) to climatic change for whole year (WY) and habitat contexts. The statistical analysis was tested by linear model. The significances were highlighted and marked with asterisks (*, *p*<0.05 and **, *p*<0.01), and (*) and ns represent *p*<0.1 and no significance respectively.

Treatments	Variables	Habitat context	AIC value	slope	*t* value	R^2	*p* value
Warming for WY	SR	MATm	235.5	-2.7	-0.4	0.01	ns
		MAP	235.6	-2.6	-0.6	0.01	ns
		AI	235.3	-1.1	-0.3	0.002	ns
	H	MATm	183.2	-10.3	-1.89	0.13	0.08 (*)
		MAP	185.9	-4.3	-0.9	0.03	ns
		AI	186.4	2.6	0.5	0.01	ns
Drought for WY	SR	MATm	147.6	-0.5	-0.2	0.001	ns
		**MAP**	**143.1**	**3.7**	**2.2**	**0.18**	**0.05***
		AI	144.8	2.3	1.7	0.11	ns
	H	MATm	80.4	-0.4	-0.1	0.01	ns
		**MAP**	**75.6**	**6.9**	**3.6**	**0.57**	**0.004****
		**AI**	**70.3**	**3.9**	**2.2**	**0.33**	**0.04***
Irrigation for WY	SR	MATm	308.9	-11.6	-1.64	0.07	ns
		**MAP**	**309.2**	**-7.7**	**-2.3**	**0.13**	**0.03***
		AI	306.5	-5.9	-1.5	0.06	ns
	H	MATm	128.7	15.5	0.3	0.01	ns
		**MAP**	**123.5**	**-42.4**	**-2.4**	**0.36**	**0.04***
		**AI**	**123.2**	**-46.3**	**-2.4**	**0.37**	**0.04***

Table S7. The differences between dry and none water-limited sites for vegetation variables to climatic experiments. The dry sites stand for the mean annual precipitation (MAP) less than 500 mm while none water-limited ones of MAP larger than 600mm. The vegetation variables of aboveground biomass (AGB), Species richness (SR) and community composition (H) between the two climatic types were separately analysed by ANOVA with Turkey HSD. The significances were highlighted and marked with asterisks (**, p<0.01), and (*) and ns represent p<0.1 and no significance respectively.

Treatments	Variables	dfHSD	*p* value
Warming for WY	SR	4	ns
	H	3.7	ns
Drought for WY	SR	-3	ns
	H	-6.1	0.07(*)
Irrigation for WY	SR	**12**	**0.005****
	H	20.7	ns

Table S8. The overall and individual trend of aboveground net primary production (ANPP) and species diversity (SR) sensitivity to climate treatments over time. The significance is tested by linear mixed model. The experiment years represent the years of the experiment while the study years are the sampling year in the study. All the regressions with significance are highlighted and marked by asterisks (*, *p*<0.05 and **, *p*<0.01), and (*) and ns represent *p*<0.1 and no significance respectively.

Treatment	Community	Code	MAP	Year	Slope	*t* value	*p* value
ANPP	**Overall**	**-**	**-**	**0-23**	**0.8**	**2.4**	**0.02***
	**1**	**Harte et al 2016**	**750**	**23 (19)**	**0.77**	**3.2**	**0.005****
	**2**	**Shi et al 2015 (Unclipp)**	**914**	**14 (14)**	**2.03**	**2.4**	**0.03***
	3	Shi et al 2015 (Clipp)	914	14 (14)	1.61	2.04	0.06(*)
	4	Collins et al 2017	250	8 (8)	-3.3	-0.6	ns
	5	Mueller et al 2016	397	7 (7)	2.35	1.04	ns
	6	Ma et al 2017	485	5 (5)	0.88	0.33	ns
	7	Cantarel et al 2013	780	5 (5)	-3.24	-1.98	ns
	8	Wang et al 2012	600	5 (5)	-1.2	-0.5	ns
	9	Henry et al 2015	260	7 (7)	-1.1	-0.24	ns
SR	Overall	-	-	0-23	-0.3	-1	ns
	1	Shi et al 2015 (Unclipp)	914	14 (14)	-0.48	-0.99	ns
	2	Shi et al 2015 (Clipp)	914	14 (14)	-1.96	-0.82	ns
	3	Hollister et al 2015	74.5	17 (4)	0.11	0.56	ns
	4	Hollister et al 2015	74.5	17 (4)	-0.08	-0.4	ns
	5	Hollister et al 2015	74.5	17 (4)	0.33	1.53	ns
	**6**	**Hollister et al 2015**	**66.6**	**19 (4)**	**-0.3**	**-25.63**	**0.002****
	7	Alatalo et al 2014	808	12 (4)	-1.2	-6.4	0.09(*)
	8	Yang et al 2017	383	9 (9)	2.67	1.51	ns
	9	Yang et al 2017	383	10 (9)	-1.06	-1.59	ns
	10	Ma et al 2017	485	5 (5)	-1.1	-1.6	ns
	11	Collins et al 2017	250	8 (8)	-0.3	-0.1	ns

Table S9. The overall and individual trend of species richness (SR) sensitivity to warming and irrigation over time. The significance is tested by linear mixed model. The experiment years represent the years of the experiment while the study years are the sampling year in the study. All the regressions with significance are highlighted and marked by asterisks (*, p<0.05 and **, p<0.01), and ns represents no significance.

Treatment	Community	Code	MAP	Year	Slope	*t* value	*p* value
ANPP	Overall	-	-	0-17	-1.3	-1.5	ns
	1	Sullivan et al 2016	2378	10 (10)	-0.65	-1.7	ns
	2	Sullivan et al 2016	2378	10 (10)	-1.47	-1.42	ns
	3	Zhu et al 2016	642.4	17 (17)	-0.5	-0.8	ns
	4	Morecroft et al 2004	818	8 (8)	-1.53	-1.04	ns
	5	Ma et al 2017	485	5 (5)	0.06	0.03	ns
	6	Collins et al 2017	250	8 (8)	-16.6	-1.7	ns
SR	**Overall**	**-**	**-**	**0-13**	**-0.9**	**-2.6**	**0.01***
	1	Sullivan et al 2016	2378	10 (10)	-0.47	-1.41	ns
	**2**	**Sullivan et al 2016**	**2378**	**10 (10)**	**-1.93**	**-3.34**	**0.009****
	**3**	**Walker et al 2003**	**700**	**13 (13)**	**-1.11**	**-3.22**	**0.008****
	4	Ma et al 2017	485	5 (5)	-1.4	-0.9	ns
	5	Collins et al 2017	250	8 (8)	-0.6	-0.2	ns

Figure legend

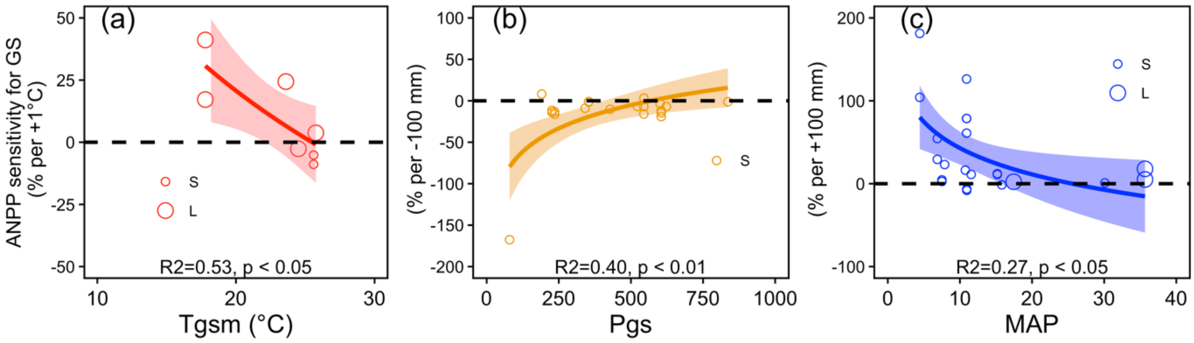

Fig. S1. The relationships between the sensitivity of ANPP and habitat contexts. The modified growing season temperature (Tgsm) (a), growing season precipitation (Pgs) (b) and mean annual precipitation (MAP) (c) were tested. The x-axis shows the best-fitted climate variables based on model selection; R^2 is the coefficient of determination for the regression. Circle sizes represent short-term (S, 1-4 years) and long-term (L, ≥5 years) studies. Statistical significance across spatial scales was tested using a general linear model. The shading with the lines indicates the standard error. The dashed line (y=0) separates positive and negative values.
